# The Global Regulator CcpA of *Listeria monocytogenes* Confers Sensitivity to Antimicrobial Fatty Acids

**DOI:** 10.3389/fmicb.2022.895942

**Published:** 2022-05-03

**Authors:** Rikke S. S. Thomasen, Magnus Ganer Jespersen, Katrine Jørgensen, Patricia T. dos Santos, Eva M. Sternkopf Lillebæk, Marianne N. Skov, Michael Kemp, Birgitte H. Kallipolitis

**Affiliations:** ^1^Department of Biochemistry and Molecular Biology, University of Southern Denmark, Odense, Denmark; ^2^Department of Microbiology and Immunology, The Peter Doherty Institute for Infection and Immunity, University of Melbourne, Melbourne, VIC, Australia; ^3^National Food Institute, Technical University of Denmark, Kgs. Lyngby, Denmark; ^4^Department of Clinical Microbiology, Odense University Hospital and Research Unit of Clinical Microbiology, University of Southern Denmark, Odense, Denmark; ^5^The Regional Department of Clinical Microbiology, Region Zealand, Zealand University Hospital, Koege, Denmark

**Keywords:** Ccpa, *Listeria monocytogenes*, antimicrobial fatty acids, anti-virulence activity, tolerance

## Abstract

Free fatty acids (FFAs) are known to exhibit antimicrobial and anti-virulent properties against bacterial pathogens. Specific FFAs, such as lauric acid (LA; C12:0), exert both effects against the foodborne pathogen *Listeria monocytogenes*: at low levels, LA acts to inhibit the activity of the virulence regulator PrfA, whereas at higher levels, LA inhibits bacterial growth. Deletion of *prfA* is known to promote tolerance toward antimicrobial FFAs, suggesting that the response of *L. monocytogenes* to anti-virulent and antimicrobial FFAs could be linked. In this study, we explored the response of *L. monocytogenes* toward antimicrobial FFAs holding an anti-virulence activity by isolating strains that can grow at high concentrations of LA. We found that LA-tolerant isolates carry mutations in the gene encoding the global regulator CcpA. Importantly, we discovered that mutation or deletion of *ccpA* protect *L. monocytogenes* against the antimicrobial activity of FFAs, whereas the *ccpA* mutants remain sensitive toward FFA’s PrfA inhibitory effect. A regulatory link involving CcpA, connecting the response toward the antimicrobial and anti-virulence activities of FFAs, is therefore unlikely. To further study how deletion of *ccpA* promotes FFA tolerance, we performed a transcriptomic analysis of the response to LA. Our data indicated that the FFA-tolerant phenotype of the ∆*ccpA* strain is not induced upon LA exposure but appears to be an inherent phenotypic trait of the *ccpA* deletion mutation. Interestingly, we found that the bacterial surface of *L. monocytogenes* becomes more hydrophilic upon deletion of *ccpA*, and we demonstrate that CcpA plays a role in the response of *L. monocytogenes* to other stress conditions, including low pH and antibiotics. Altogether, our study revealed that regulatory activities of CcpA lead to an increased hydrophobicity of the bacterial surface, which may confer sensitivity of *L. monocytogenes* against the antimicrobial activity of FFAs. Notably, CcpA is not involved in responding to the PrfA inhibitory effect of FFAs, showing that FFA-tolerant strains can still be targeted by the anti-virulent activity of FFAs.

## Introduction

*Listeria monocytogenes* is a Gram-positive foodborne bacterium causing life-threatening infections in humans and animals. During infection of susceptible individuals, *L. monocytogenes* gains access to the cytoplasm of host cells; here, the bacterium multiplies and spreads from cell-to-cell through a mechanism that involves host actin polymerization (Freitag et al., 2009). Several virulence factors are known to contribute to the intracellular lifestyle of *L. monocytogenes*, including the internalins InlA and InlB, which allow bacterial invasion of non-phagocytic cells; the pore-forming toxin LLO, which is required for escape from host cell vacuoles, and the surface protein ActA, which promotes actin polymerization and cell-to-cell movement (Freitag et al., 2009). Inside the intracellular environment, the virulence regulator, PrfA, responds to bacterial- and host-derived glutathione (GSH) and activates transcription of PrfA-regulated virulence genes encoding LLO, ActA, and other virulence factors required for intracellular infection (Scortti et al., 2007; de las Heras et al., 2011; Reniere et al., 2015; Hall et al., 2016). In the extracellular environment, PrfA is generally not active, but constitutively active mutant variants of PrfA, named PrfA*, are known to bypass the need of GSH for PrfA activation of virulence gene expression (Ripio et al., 1997b; Eiting et al., 2005; Xayarath and Freitag, 2012; Johansson and Freitag, 2019). PrfA* proteins are locked in an active conformation that promotes optimal binding to specific DNA sequences (PrfA boxes) located in the promoter regions of PrfA-regulated virulence genes (Eiting et al., 2005; Johansson and Freitag, 2019).

Upon ingestion of contaminated food, *L. monocytogenes* enters the gastrointestinal tract. Here, the pathogen encounters dietary components, the gut microbiota, and host immune parameters. Together, these conditions strongly influence the ability of *L. monocytogenes* to cause disease (Tiensuu et al., 2019). We previously found that specific dietary free fatty acids (FFAs) act as signaling molecules to reduce virulence factor expression in *L. monocytogenes* by a mechanism that involves direct inhibition of PrfA (Kallipolitis, 2017; Sternkopf Lillebæk et al., 2017; Dos Santos et al., 2020). Interestingly, exposure to specific FFAs prevented the constitutively active variant PrfA* from binding to the PrfA box in the promoter region of *hly*, encoding LLO (Dos Santos et al., 2020). Notably, some PrfA inhibitory FFAs also exert an antimicrobial effect on *L. monocytogenes* (Sternkopf Lillebæk et al., 2017; Dos Santos et al., 2020). The mechanism underlying the antimicrobial activity of FFAs is presently unknown, but they most likely target and interfere with vital functions of the bacterial membrane (Desbois and Smith, 2010).

The saturated medium-chain fatty acid lauric acid (LA; C12:0) is commonly found in nuts, seeds, plants, and milk and is generally known as a potent antimicrobial agent (Petrone et al., 1998; Desbois and Smith, 2010). Growth of *L. monocytogenes* is efficiently inhibited in the presence of 50 μg/ml LA in rich medium, whereas at subinhibitory concentrations (≤10 μg/ml), LA acts to inhibit PrfA-dependent activities (Sternkopf Lillebæk et al., 2017). Thus, LA belongs to the category of dietary FFAs that act as an antimicrobial agent as well as a virulence inhibitory signaling compound in *L. monocytogenes*. We previously observed that the general stress sigma factor, Sigma B, is dispensable for the tolerance of *L. monocytogenes* against FFAs, suggesting that the response of *L. monocytogenes* to antimicrobial FFAs relies on other stress regulatory pathways (Sternkopf Lillebæk et al., 2017). Curiously, a Δ*prfA* mutant strain grows well in the presence of >75 μg/ml LA, suggesting that PrfA somehow acts to increase the sensitivity of *L. monocytogenes* to the antimicrobial activity of LA (Sternkopf Lillebæk et al., 2017). This means that PrfA could play several roles in the response to LA: at subinhibitory concentrations, LA targets PrfA directly to inhibit its DNA binding activity, resulting in repression of key virulence genes, whereas at higher concentrations, LA relies on PrfA for its antimicrobial activity (Sternkopf Lillebæk et al., 2017; Dos Santos et al., 2020). These findings prompted us to investigate in more detail the molecular mechanisms underlying the response of *L. monocytogenes* to LA.

In the present study, we aimed to reveal if the anti-virulence activity of LA can be linked to its antimicrobial action. To address this question, we isolated LA-tolerant *L. monocytogenes* mutant strains and analyzed their response to the PrfA inhibitory signaling effect of LA. Curiously, the LA-tolerant strains expressed a mutant version of the catabolite control protein A, CcpA, containing an extended C-terminal tail. CcpA is known as the major global transcription regulator of carbon catabolite repression (CCR) in *L. monocytogenes* and other Gram-positive bacteria; a complex regulatory mechanism that allows bacteria to use available carbon sources in an optimal manner (Jones et al., 1997; Herro et al., 2005; Deutscher, 2008). Accordingly, CcpA acts to repress multiple genes encoding proteins involved in transport and metabolism of various carbohydrates (Mertins et al., 2007) Here, we present the results of our investigations on the role of CcpA in the response of *L. monocytogenes* to the antimicrobial and PrfA inhibitory FFA, LA.

## Materials and Methods

### Bacterial Strains and Growth Conditions

In this study, we used a wild-type *L. monocytogenes* EGD serotype 1/2a strain and its isogenic mutant derivative Δ*prfA* (Böckmann et al., 1996) obtained from W. Goebel (Biozentrum). Furthermore, we used the isogenic mutant strain EGD-prfA* expressing the constitutively active PrfA mutant derivative G155S; this strain was constructed in a previous study (Sternkopf Lillebæk et al., 2017). As part of the present study, the EGD-prfA* strain (from here, prfA*) was genome sequenced and compared to the genomes of the EGD and EGD-e strains sequenced by Bécavin et al. (2014). A single nucleotide polymorphism (SNP) search revealed 20101 SNPs between prfA* and the EGD strain studied by Bécavin et al. (2014), whereas only 10 SNPs were found when comparing prfA* and EGD-e. These findings showed that the EGD derivatives used in the present study are more closely related to EGD-e than the EGD strain sequenced by Bécavin et al. (2014). We therefore used EGD-e as reference genome for the whole-genome sequencing and RNA-sequencing analyses (described below).

For construction of the *ccpA*-mut1 1 bp deletion (A_8 → 7_ at location 1,642,875), in-frame deletion of 981 bp in *ccpA* (Δ*ccpA*); 1955 bp in *lmo0109-0110* (∆*lmo0109-0110*); 792 bp in *lmo0517* (∆*lmo0517*); 744 bp in *lmo2175* (∆*lmo2175*); and 1830 bp in *lmo2772* (∆*lmo2772*), the corresponding primers P1, P2, P3 and P4 (Supplementary Table S7) were used, respectively, for a 2-step PCR amplification of the fragments. CcpA complementation mutant (prfA*-∆*ccpA::*compl.) was constructed using P1 and P4 for ∆*ccpA* and the fragment was produced by a 1-step PCR reaction. The fragments were inserted into the temperature sensitive shuttle vector pAUL-A (Schäferkordt and Chakraborty, 1995) and transformed into *L. monocytogenes* as earlier described (Mollerup et al., 2016). Homologous recombination was carried out as described previously (Christiansen et al., 2004). The resulting deletion mutants were validated by PCR using primers P5 and P6. The plasmids p*hly-lacZ*, with a transcriptional fusion between the *hly* promoter and the *lacZ* gene, and p*lhrA-36-lacZ*, containing a transcriptional fusion between the *lhrA* core promoter and *lacZ*, were constructed previously (Larsen et al., 2006; Nielsen et al., 2011). *L. monocytogenes* was routinely grown in brain heart infusion broth (BHI, Oxoid) at 37°C with aeration. When appropriate, cultures were supplemented with either kanamycin (50 μg/ml) or erythromycin (5 μg/ml). During cloning in pAUL-A, *Escherichia coli* TOP10 (Invitrogen) was grown in Luria-Bertani broth (LB, Sigma) supplemented with 150 μg/ml erythromycin at 37°C with aeration.

### Fatty Acid-Tolerant Strains

Fatty acids used in this study were lauric acid (LA; C12:0; Sigma-Aldrich, purity ≥98%), palmitoleic acid (PA; C16:1; Sigma-Aldrich, purity ≥98.5%), and palmitic acid (PAL; C16:0; Sigma-Aldrich, purity ≥99%). 96% ethanol was used as vehicle to dissolve the FFAs.

Three independent ON cultures of prfA* were diluted to OD_600_ = 0.0002 and stressed with increasing concentrations of LA (10 μg/ml, 20 μg/ml, 40 μg/ml, 80 μg/ml, 160 μg/ml, 320 μg/ml, and 500 μg/ml) for 7 days; the concentration of vehicle was kept constant at 0.25% during the selection process. Glycerol stocks were made, and single mutants were isolated from the three biological replicates. Bacterial identification was performed by PCR using primers for *hly* (Supplementary Table S7).

### Growth Experiments

For growth experiments in culture flasks, ON cultures were diluted to OD_600_ = 0.002. Growth was monitored until cultures reached stationary phase by OD_600_ measurements.

In growth experiments, where strains were screened for FFA tolerance, ON cultures were diluted to OD_600_ = 0.0002 and 4 ml was transferred to glass tubes with various concentrations of LA, PA, or PAL. As controls, cultures were left untreated or stressed with vehicle corresponding to the highest concentration used in FFA-treated samples. OD_600_ was measured after 20 h of incubation.

Growth experiments with other stress conditions than FFAs were performed in a plate reader (Synergy^™^ H1 multi-mode microplate reader, BioTek) using 96-well plates (standard, F, SARSTEDT). ON cultures were diluted to a final OD_600_ = 0.005 in the 96-well plate with the different stress conditions. The plate was incubated at 37°C with 15 s. of orbitally shaking every 30 min for 24 h.

### β-Galactosidase Assays

ON cultures of the strains containing the plasmids p*hly-lacZ* or p*lhrA-36-lacZ* were diluted to OD_600_ = 0.02. At OD_600_ = 0.3 the cultures were split and FFA was added to the following final concentrations: 10 μg/ml LA, 2 μg/ml PA, or 150 μg/ml PAL. As control, vehicle was added corresponding to the final concentration in FFA-treated cultures. Samples (1 ml) were harvested after 20 h of growth. β-galactosidase assay was conducted as previously described (Christiansen et al., 2004).

### Whole-Genome Sequencing

Sequencing libraries of prfA* and the nine isolated LA-tolerant strains were prepared using Nextera XT DNA kit. Libraries were sequenced using Illumina Miseq platform in pair-end mode, read length of 150 bp. The quality of the reads was tested using FastQC standard settings. Before SNP analysis, the reads were trimmed using seqTK. The trimmed reads were mapped and polymorphisms called relative to the reference genome of *L. monocytogenes* EGD-e (NCBI ASM19603v1) using Breseq standard settings (Deatherage and Barrick, 2014). SNPs common for both read directions were found using gdtools and are listed in Table 1.

**Table 1:** Mutations found in LA-tolerant strains by WGS.

LA-tolerant strains	Location	Gene	Codon	Mutation	Description
^1^LA-1A, LA-1D, LA-2A, LA-2B LA-3A, LA-3B, LA-3C	1,642,875	*ccpA*	333	$A_{8\to 7}$	1 bp is deleted resulting in a frameshift that removes the STOP codon. The new STOP codon is therefore placed 26 codons downstream *ccpA*.
LA-1B	1,642,965	*ccpA*	303	G → T	Nucleotide substitution, which results in a missense mutation. Arginine is substituted by leucine (R303L).
LA-1C	1,033,670	*lmo1003*	161	${\left(\mathrm{TTG}\right)}_{3\to 4}$	In-frame mutation, where an extra codon is inserted after codon 160, which extends the repeat sequence ${\left(\mathrm{TTG}\right)}_{3\to 4}$ , and results in addition of an extra leucine amino acid into the protein sequence.

1 The strains ccpA-mut1 and prfA*-ccpA-mut1 were constructed by site-directed mutagenesis to contain the frameshift mutation observed in these LA-tolerant strains.

### Total RNA Extraction and Purification

ON cultures of prfA* and prfA*-Δ*ccpA* were diluted in BHI medium to OD_600_ = 0.02. At OD_600_ = 0.35 cultures were split and left untreated or treated with a final concentration of 10 μg/ml LA. After 1 h, 5 ml samples were mixed with 10 ml RNAprotect bacteria reagent (Qiagen) and incubated at RT for 5 min. The samples were centrifuged at 8,000 rpm for 3 min at 4°C, and pellet was snap-cooled in liquid nitrogen.

Cells were disrupted by the Fastprep instrument (Bio101, Thermo Scientific Corporation). Total RNA was extracted by Tri reagent (Molecular Research Center, Inc.), as previously reported (Nielsen et al., 2010). RNA purity and concentration were determined by agarose gel electrophoresis and DeNovix DS-11 Fx.

### Northern Blot Analysis

Agarose northern blot analysis was performed as described previously (Dos Santos et al., 2020). The membrane was hybridized with ^32^P-labeled single-stranded probes (Supplementary Table S7). Visualization of bands was performed using Typhoon FLA9000 (GE Healthcare) and quantified using IQTL 8.0 quantification software (GE Healthcare).

### rRNA Removal

To remove rRNA, RiboMinus^™^ Transcriptome Isolation Kit (Yeast and Bacteria; Invitrogen) was used. Briefly, magnetic beads were washed twice in RNase-free water, once in hybridization buffer, and then resuspended in hybridization buffer and kept at 37°C until use. A total of 8 μg RNA were incubated with RiboMinus probe and hybridization buffer at 37°C for 5 min to denature RNA, and samples were incubated on ice for 30 s. Cooled hybridized samples were mixed with the magnetic beads and incubated for 15 min at 37°C. The supernatant was isolated, and mRNA was precipitated by ethanol precipitation. 1 μl glycogen (20 μg/μL), 0.1X sample volume of 3 M sodium acetate and 2.5X sample volume of 96% ethanol was added to the supernatant. Samples were incubated at −20°C for 50 min. Precipitated mRNA was washed twice in 70% cold ethanol and resuspended in RNase-free water after the pellet was air-dried.

### Library Preparation, RNA-Sequencing, and Analysis

Libraries were constructed utilizing the NEBNext Ultra RNA Library Prep Kit for Illumina according to the manufacture’s protocol (NEB) and paired-end sequenced on the NovaSeq 6,000 platform (Illumina). The quality of the sequenced reads was checked by FastQC. Reads were mapped to the reference genome of EGD-e (NCBI ASM19603v1) using Bowtie 2 version 2.3.5.1 with standard settings and local alignment (Langmead and Salzberg, 2012). SAM files were converted to BAM files, sorted, and indexed by samtools version 1.7. Sorted BAM files were loaded as input in SeqMonk mapped sequence data analyzer version 1.45.3 with following settings: Duplicate reads were not removed, the minimum mapping quality was set to 28, primary alignments only, and paired-end RNA-seq data. Raw counts were generated by RNA quantification pipeline. Differentially expressed (DE) genes were found by DESeq2 analysis using R version 4.0.3 and were reported as log_2_ fold changes. Fold changes were calculated in this study by comparing expression levels for LA vs. control condition for prfA*, and prfA*-∆*ccpA* (Supplementary Tables S2, S3, respectively). Additionally, expression level of prfA*-∆*ccpA* vs. prfA* upon control and LA conditions were compared (Supplementary Tables S4, S5, respectively). Genes that were found to have a log_2_ ≥ 1 or ≤ −1 (at least a 2-fold change) and a *value of p* below 0.05 were determined to be DE genes. Genes with less than 10 raw reads in all six biological replicates included in the comparison were excluded.

### Hydrophobicity Assay

Bacteria were harvested from ON cultures by centrifugation for 5 min at 4,000 rpm and supernatant was removed. Bacteria were washed three times in 5 ml 1 × PBS and diluted in 1 x PBS to OD_600_ = 0.3 (OD_600__1). 300 μl n-hexadecane (Sigma-Aldrich) was added to 3 ml of the diluted cultures in culture tubes and samples were vortexed for 2 min followed by 15 min incubation at RT for phase separation. Afterward, OD_600_ was measured again for the water phase (OD_600__2). Percentage of cells staying in the hydrophilic phase was calculated by OD_600__2/OD_600__1 × 100%. Data were analyzed using two-tailed t-test. Only differences with at least 95% confidence were reported as statistically significant.

## Results

### Selection of LA-Tolerant Strains

**Figure 1:**
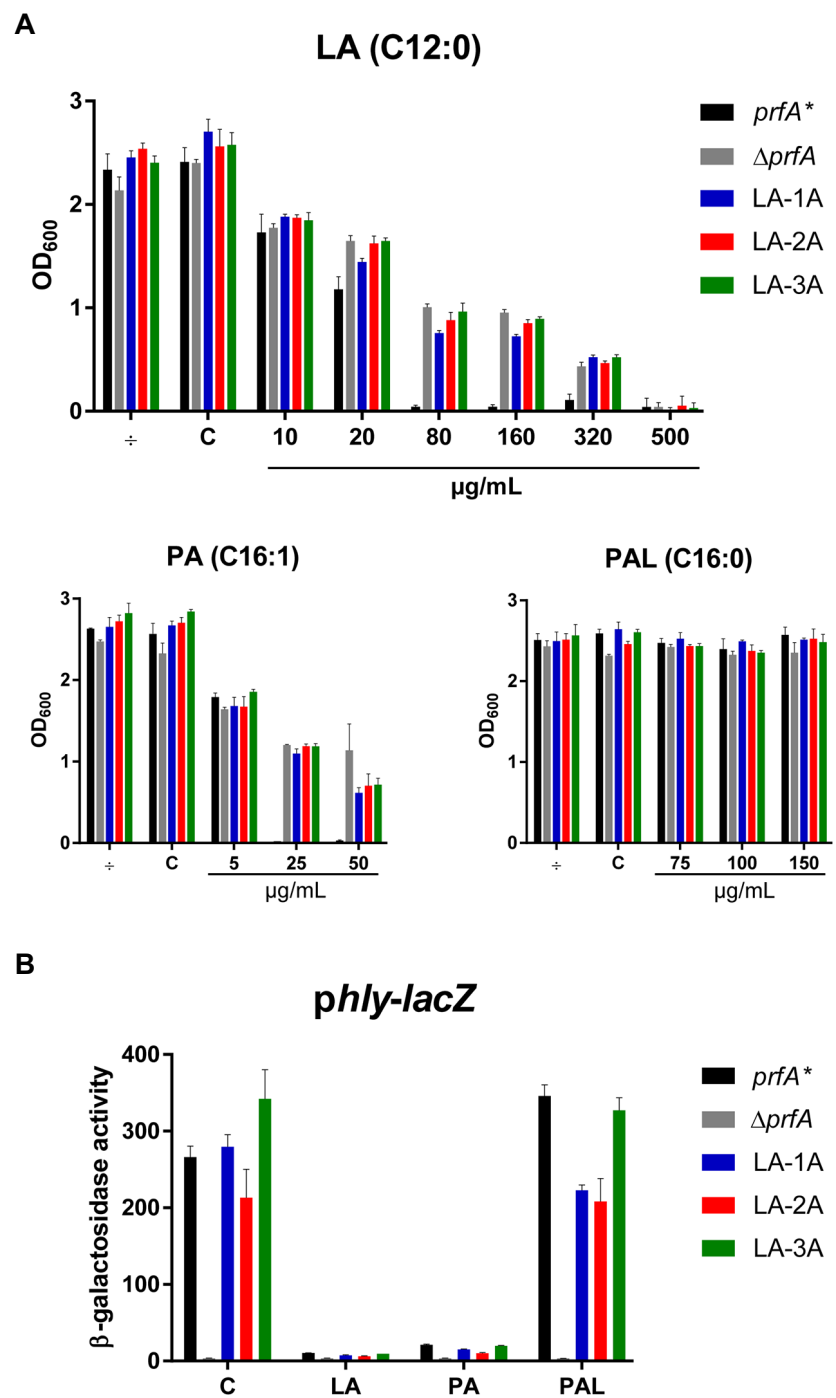
The response of selected LA-tolerant strains toward antimicrobial and anti-virulent FFAs. **(A)** Growth of isolated LA-tolerant strains ON. The prfA*, ∆*prfA* and one isolate from each biological replicate of the selected LA-tolerant strains (LA-1A, LA-2A, and LA-3A) were diluted to OD_600_ = 0.0002 and exposed to various concentrations of LA, PA, or PAL. As controls, cultures were left untreated (÷) or exposed to a corresponding concentration of vehicle (C). Growth was measured after 20 h. Results are the average of at least three independent experiments. **(B)** β-galactosidase assay for LA-tolerant strains. The pTCV-*lacZ*-derivative containing the *hly* promoter fused to *lacZ* was transformed into prfA*, ∆*prfA*, and selected LA-tolerant strains (LA-1A, LA-2A, and LA-3A). The resulting strains were grown to OD_600_ = 0.3 and exposed to 10 μg/ml LA, 2 μg/ml PA, or 150 μg/ml PAL. As control, strains were incubated with a corresponding concentration of vehicle (C). Bacteria were harvested after 20 h of growth. Results are the average of three independent experiments, each performed in technical duplicates.

To generate FFA-tolerant strains, L. monocytogenes prfA* was grown in BHI medium containing increasing concentrations of the antimicrobial and PrfA inhibitory FFA, LA. LA-tolerant strains were selected in a prfA* background to allow further studies on the PrfA inhibitory activity of FFAs in BHI medium (Sternkopf Lillebæk et al., 2017; Dos Santos et al., 2020). A total of nine single strains were isolated from three independent biological replicates grown with 500 μg/ml LA. To test if these isolates had obtained tolerance toward LA, they were grown in the presence of increasing concentrations of LA (Figure 1A; Supplementary Figure S1). In addition to the parental strain, a PrfA-deficient strain, Δ*prfA*, was included as control since deletion of *prfA* is known to increase the tolerance of *L. monocytogenes* to antimicrobial FFAs (Sternkopf Lillebæk et al., 2017). All nine isolates and Δ*prfA* could grow at 6-fold higher concentrations of LA relative to the parental strain, prfA*, demonstrating that the selected strains were indeed tolerant toward LA (Figure 1A; Supplementary Figure S1). To investigate if the strains selected were tolerant to other FFAs, growth experiments were performed with the antimicrobial and PrfA inhibitory FFA palmitoleic acid (PA; C16:1). As control, we included its saturated counterpart palmitic acid (PAL; C16:0), which is known to leave *L. monocytogenes* unaffected (Sternkopf Lillebæk et al., 2017). Interestingly, the nine selected strains showed an increased tolerance toward PA as well, whereas their response to PAL was comparable to the parental strain (Figure 1A; Supplementary Figure S1). Thus, although the strains were selected for their LA-tolerant phenotype, they were clearly tolerant to the antimicrobial FFA PA as well.

### The Inhibitory Effect of FFAs on PrfA Is Unaffected by FFA Tolerance

We previously showed that LA and PA act to inhibit PrfA-dependent activation of virulence genes in L. monocytogenes prfA* (Sternkopf Lillebæk et al., 2017). Importantly, the PrfA inhibitory activities were observed at subinhibitory concentrations of LA and PA. To study how the FFA-tolerant strains respond to the PrfA inhibitory activity of LA and PA, a β-galactosidase assay was performed. Briefly, the nine selected strains were transformed with a p*hly-lacZ* fusion plasmid, which contains the PrfA-activated *hly* promoter fused to the reporter gene *lacZ* in the vector pTCV-lac (Larsen et al., 2006). Furthermore, the strains were transformed with the control reporter plasmid p*lhrA-*36-*lacZ*, which contains a PrfA-independent promoter fused to *lacZ*. Again, prfA* and Δ*prfA* were included as controls. The resulting strains were exposed to subinhibitory levels of LA, PA, PAL, or vehicle, and cells were harvested after 20 h of growth. Under control conditions, all strains (except from Δ*prfA*) produced high levels of β-galactosidase activity, showing that the nine selected strains encode functional PrfA* protein (Figure 1B and Supplementary Figure S2). Exposure to the non-inhibitory PAL did not affect the promoter activity of *hly* in any of the strains tested (Figure 1B; Supplementary Figure S2A). In contrast, the activity was clearly repressed in prfA* and the nine tolerant strains upon exposure to LA or PA (Figure 1B, Supplementary Figure S2A). Notably, all strains containing the PrfA-independent reporter plasmid p*lhrA-36-lacZ* were largely unaffected by the FFAs (Supplementary Figure S2B). These results demonstrate that LA and PA act to inhibit PrfA-dependent activation of *hly* in the nine selected strains, even at FFA levels much lower (approx. 50-fold) than required for exerting a growth inhibitory response.

Altogether, these results suggest that all nine LA-tolerant strains encode functional PrfA protein, implying that the FFA-tolerant phenotype is likely due to mutations in genes other than *prfA*. Furthermore, the PrfA inhibitory effect of LA and PA appears to be unaffected by the FFA-tolerant phenotype of the selected strains.

### A Frameshift Mutation or Deletion of *ccpA* Result in FFA Tolerance

**Figure 2:**
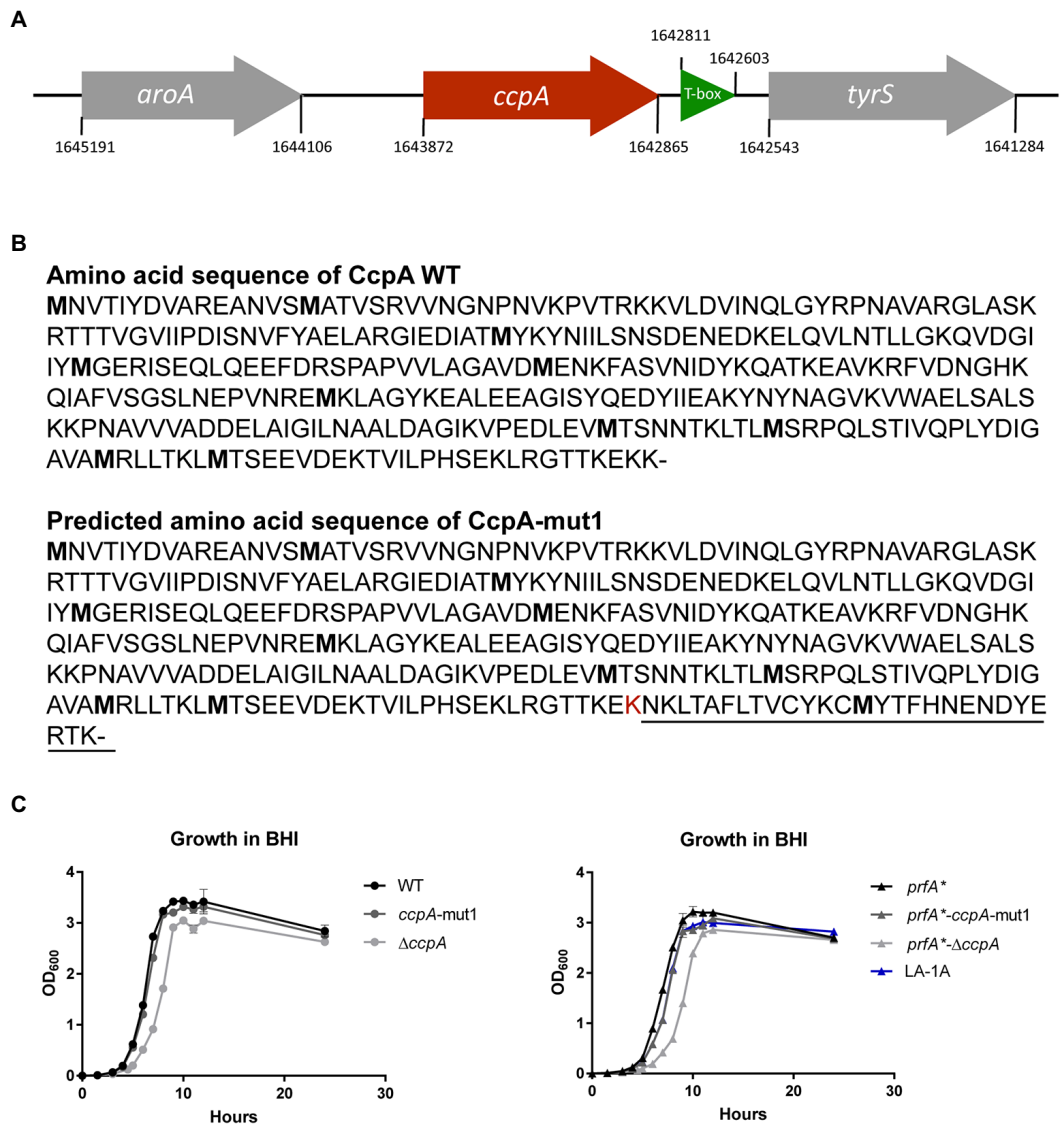
LA-tolerant strains carry mutations in the *ccpA* gene **(A)** Schematic illustration of the genomic region encoding CcpA. The *aroA* gene (gray) is located upstream from *ccpA* (red), whereas *tyrS* (gray) is situated downstream from *ccpA*. Potentially, mutations in *ccpA* could have polar effects on the expression of *tyrS*, which is regulated *via* an upstream located T-box leader, shown in green. However, in our RNA-seq analyses, we did not observe any significant differences in *tyrS* expression when comparing strains prfA* and prfA*-Δ*ccpA* (this study) or strains prfA* and prfA*-ccpA-mut1 (our unpublished observations). **(B)** The annotated amino acid sequence of wild-type CcpA protein and the predicted CcpA-mut1 amino acid sequence. The estimated amino acid sequence of CcpA-mut1 is based on the frameshift mutation detected by whole-genome sequencing and terminator predictions using the web tool ARNold finding terminators. **(C)** Growth of CcpA mutants in BHI medium. Cultures of wild-type, *ccpA*-mut1, ∆*ccpA*, prfA*, prfA*-ccpA-mut1, prfA*-∆*ccpA*, and LA-1A were diluted to OD_600_ = 0.002. Growth was measured every hour for the first 12 h with an additional 24 h point. Results are the average of three independent experiments.

The nine LA-tolerant strains were characterized through whole-genome sequencing. Three mutations were found in the nine selected strains: a frameshift mutation in *ccpA*, a missense mutation in *ccpA* or an in-frame mutation in *lmo1003* (Table 1). The frameshift mutation in *ccpA* was consistently found in seven out of nine strains; notably, this mutation was represented in strains selected from all three biological replicates (Table 1). The genomic organization of the region encoding *ccpA* is illustrated in Figure 2A. In *L. monocytogenes*, CcpA is known as a major transcriptional regulator of carbon metabolism (Behari and Youngman, 1998; Mertins et al., 2007). The activity of CcpA is modulated by different cofactors, and it controls the expression of many genes, most importantly those involved in the uptake and metabolism of carbohydrates (Galinier et al., 1997). CcpA consists of a N-terminal DNA binding domain and a C-terminal core domain involved in cofactor binding (Chaptal et al., 2006). Most likely, the 1 bp deletion in codon 333 (of 336) results in a CcpA protein, from now on referred to as CcpA-mut1, with an extended C-terminus (Figure 2B).

Since the *ccpA-*mut1 frameshift mutation is highly prevalent among the LA-tolerant strains, we decided to specifically examine the effect of *ccpA*-mut1 on FFA tolerance. Thus, the *ccpA*-mut1 frameshift mutation was introduced in the parental strain, prfA*, and the wild-type strain. Furthermore, since the expected extension of the CcpA protein in *ccpA*-mut1 could affect the protein’s functionality, we constructed *ccpA* knock-out strains, corresponding to an in-frame deletion of the entire *ccpA* gene in the prfA* and wild-type strains, allowing a direct comparison of *ccpA* phenotypes. First, the effect of *ccpA-*mut1 and Δ*ccpA* on bacterial growth was compared under standard growth conditions (Figure 2C). For prfA* and wild-type, we observed that *ccpA*-mut1 had a minor effect on growth in BHI medium (2–8% increase in doubling time, Supplementary Table S1). Furthermore, comparable doubling times were determined for prfA*-*ccpA*-mut1 and one of the selected strains harboring the *ccpA*-mut1 mutation (LA-1A). Notably, deletion of *ccpA* had a clear effect on the bacterial growth (Figure 2C); the doubling time of the Δ*ccpA* mutant strains increased by 22–36% compared to the parental strains (Supplementary Table S1). The growth phenotype of Δ*ccpA* was restored to that of the parental strains by complementation with wild-type *ccpA* (Supplementary Figure S3A). Similar growth phenotypes were obtained in a previous study, using a *ccpA* insertion mutant (Mertins et al., 2007). Collectively, our data show that deletion of *ccpA* had a more negative impact on bacterial fitness relative to the *ccpA*-mut1 mutation, suggesting that *ccpA*-mut1 frameshift mutation might not lead to complete loss of CcpA functionality.

**Figure 3:**
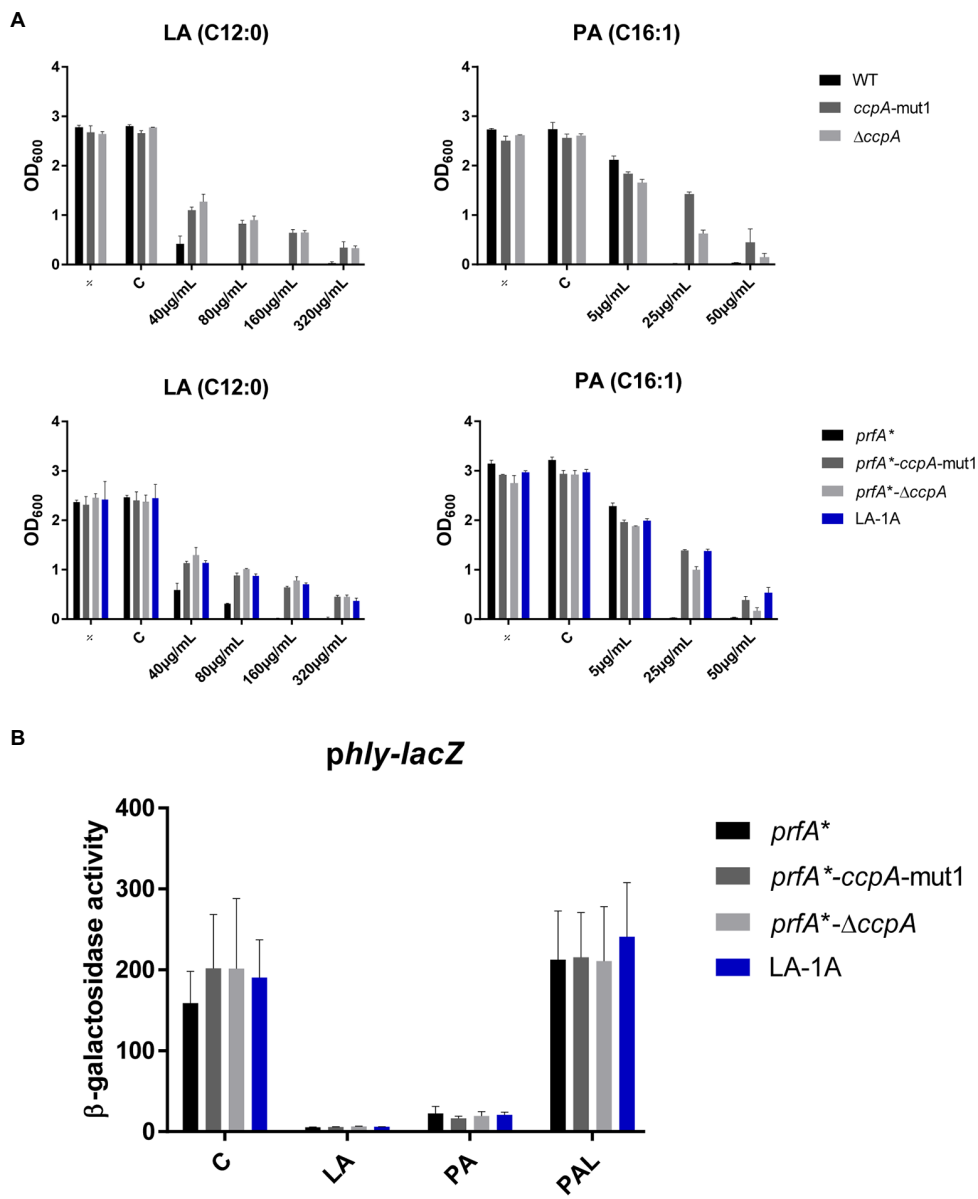
The response of CcpA mutants to antimicrobial and anti-virulent FFAs. **(A)** Growth of CcpA mutants upon LA exposure. Wild-type, *ccpA*-mut1, ∆*ccpA*, prfA*, prfA*-ccpA-mut1, prfA*-∆*ccpA* and LA-1A were diluted to OD_600_ = 0.0002 and exposed to different concentrations of LA and PA. As controls, cultures were left untreated (÷) or exposed to a corresponding concentration of vehicle (C). After 20 h of incubation growth was measured. The results represent the average of three independent experiments. **(B)** β-galactosidase assay for CcpA mutants. Strains prfA*, prfA*-ccpA-mut1, prfA*-∆*ccpA*, and LA-1A were transformed with p*hly-lacZ*, containing a transcriptional fusion of the *hly* promoter to the *lacZ* gene. Resulting strains were grown to OD_600_ = 0.3 and exposed to 10 μg/ml LA, 2 μg/ml PA, or 150 μg/ml PAL. As control, cultures were exposed to a corresponding concentration of vehicle (C). Results are the average of three independent experiments each performed in technical duplicates.

Next, the effect of *ccpA*-mut1 and Δ*ccpA* on FFA tolerance was tested by comparing the growth of mutants and parental strains in increasing concentrations of LA or PA (Figure 3A). The *ccpA*-mut1 and Δ*ccpA* mutant strains were equally tolerant toward LA and PA, and much more tolerant compared to the corresponding parental strains (Figure 3A). Complementation with wild-type *ccpA* restored the phenotype to that of the parental strain, confirming that CcpA confers sensitivity toward antimicrobial FFAs (Supplementary Figure S3B). Altogether, these data revealed that the frameshift mutation *ccpA*-mut1, present in seven out of nine selected mutants, confer FFA tolerance. Notably, Δ*ccpA* mutants were clearly tolerant to LA and PA as well, confirming that regulatory activities of CcpA confer increased sensitivity of *L. monocytogenes* to antimicrobial FFAs. Furthermore, we observed that mutation of *ccpA* confers FFA tolerance independently of PrfA activity, since an increased tolerance to LA and PA was observed in both the wild-type and the prfA* background (Figure 3A).

To examine if *ccpA*-mut1 or deletion of *ccpA* interfere with the PrfA inhibitory activity of FFAs, we performed a β-galactosidase assay. The prfA* mutant series, containing p*hly-lacZ* or p*lhrA-36-lacZ* fusion plasmids, were exposed to subinhibitory levels of LA, PA, or PAL (Figure 3B and Supplementary Figure S4). Under control conditions, *ccpA*-mut1 and Δ*ccpA* mutant cells produced high levels of β-galactosidase activity, indicating that the *ccpA* mutations *per se* do not interfere with PrfA-dependent activation of *hly*. Importantly, *ccpA*-mut1 and Δ*ccpA* mutant cells were still sensitive toward the PrfA inhibitory effect of LA and PA, since the promoter activity of *hly* was strongly reduced upon LA and PA exposure (Figure 3B). As expected, PAL had no major effect on any of the strains tested (Figure 3B). These results show that the *ccpA* mutations do not affect the activity of PrfA*; furthermore, they do not interfere with the PrfA inhibitory effect of LA and PA.

Altogether, these data clearly demonstrate that CcpA confers sensitivity toward the antimicrobial activity of the FFAs. Importantly, the PrfA inhibitory activity of the FFAs does not rely on a functional CcpA.

### Exploring How Lack of CcpA Confers FFA Tolerance

Since the *ccpA*-mut1 and Δ*ccpA* strains were equally tolerant toward FFAs (Figure 3A), we reasoned that the mechanism conferring FFA tolerance is related to the absence of CcpA’s regulatory activities. To investigate in more detail how lack of CcpA functionality confers FFA tolerance, transcriptome analysis was performed for prfA* and prfA*-∆*ccpA* under control conditions and upon exposure to a subinhibitory level LA for 1 h. Again, a prfA* background was chosen to reveal any effect of LA and/or CcpA on PrfA-dependent regulatory activities in BHI medium.

**Figure 4:**
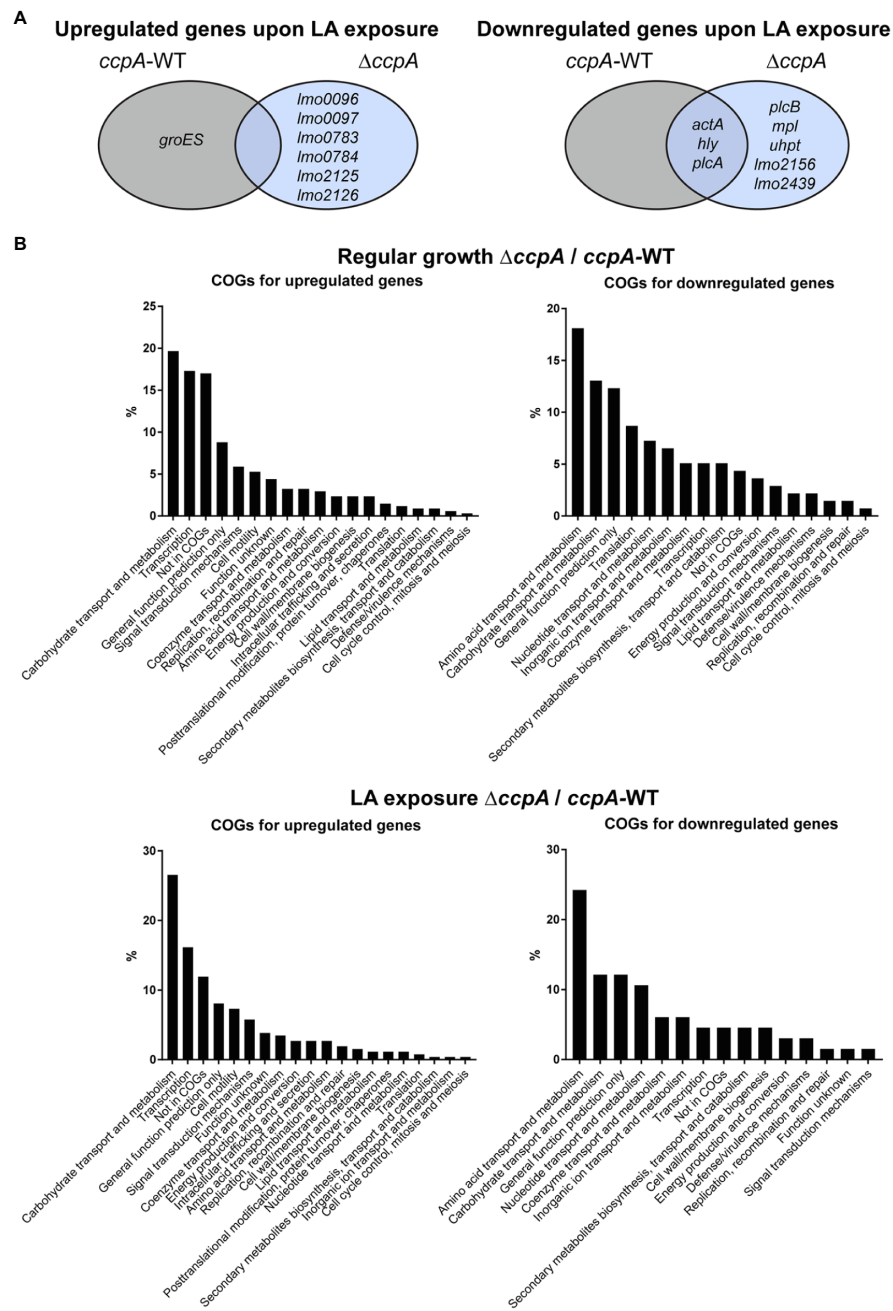
Transcriptomic profiles for prfA* and prfA*-∆*ccpA* cells upon regular growth and exposure to LA. **(A)** Venn diagram for genes significantly regulated upon exposure to LA in prfA* and prfA*-∆*ccpA*. Genes up- or downregulated by at least 2-fold upon exposure to LA relative to control conditions are gathered in a Veen diagram for prfA* and prfA*-∆*ccpA*. **(B)** Clusters of Orthologous Genes (COGs) distribution of significantly regulated genes in the ∆*ccpA* strain compared to the parental strain. The transcriptome profile of prfA*-∆*ccpA* and prfA* strains were compared both upon control conditions and upon exposure to LA. Genes significantly up- or downregulated by more than 2-fold were determined as differentially expressed. The distribution of regulated genes according to their corresponding COGs is presented in the graphs.

After RNA-sequencing, we first analyzed the response of each of the two strains to LA exposure. In general, LA seemed to have a very limited effect on global gene expression; for each strain, less than 15 genes were significantly induced or repressed by at least 2-fold in response to LA (Supplementary Tables S2, S3). The two strains do not share any of the upregulated genes (Figure 4A). For the downregulated genes, a more common tendency was observed; most importantly, the PrfA-regulated genes *hly, plcA*, and *actA* were significantly downregulated in both strains upon LA exposure (Figure 4A; Supplementary Tables S2, S3). In addition, *plcB, mpl*, and *uhpT*, which are controlled by PrfA as well, were significantly downregulated upon LA exposure in ∆*ccpA* (Figure 4A; Supplementary Tables S2, S3). To summarize, the results of the transcriptome study confirmed that LA exposure leads to downregulation of PrfA-dependent virulence gene expression in a strain encoding the constitutively active variant of PrfA, PrfA-G155S. In general, LA exposure affected only a limited set of genes in L. monocytogenes prfA*, suggesting that at subinhibitory concentrations, LA primarily acts as a signal to downregulate PrfA-dependent virulence gene expression. Additionally, we note that CcpA does not seem to be required for LA-mediated inhibition of PrfA-regulated genes.

As a very low number of genes were affected by LA exposure, we speculated that the FFA-tolerant phenotype observed for ∆*ccpA* might result from transcriptional changes caused by lack of CcpA functionality in general. Therefore, to find genes potentially important for the FFA-tolerant phenotype, we compared the transcriptomes of prfA* and prfA*-∆*ccpA* cells. Deletion of *ccpA* resulted in up- or downregulation of 312 and 143 genes, respectively, by ≥2-fold under regular growth conditions (Supplementary Table S4). As expected, more genes were up- than downregulated, because the majority of genes belonging to the CcpA regulon in *L. monocytogenes* are repressed by CcpA (Mertins et al., 2007). In accordance with CcpA’s role in CCR control, 20% of the genes upregulated in the Δ*ccpA* mutant strain are related to carbohydrate transport and metabolism (Figure 4B). The largest group of genes downregulated in prfA*-Δ*ccpA*, relative to prfA*, are involved in amino acid transport and metabolism (18%). In the presence of LA, 218 and 66 genes were significantly up- or downregulated, respectively, by ≥2-fold in the ∆*ccpA* mutant relative to the parental strain (Supplementary Table S5). The largest groups of up- and downregulated genes are still involved in carbohydrate transport and metabolism (26%) and amino acid transport and metabolism (24%), respectively (Figure 4B). The transcriptome analysis was validated by northern blot analysis of four selected genes (Supplementary Figure S5). Altogether, these results confirm that CcpA is a major gene regulator of metabolic pathways in *L. monocytogenes*, as shown previously by others (Mertins et al., 2007). Notably, although CcpA promotes sensitivity to antimicrobial FFAs, its regulatory activities appear to be largely unaffected by FFA exposure.

**Figure 5:**
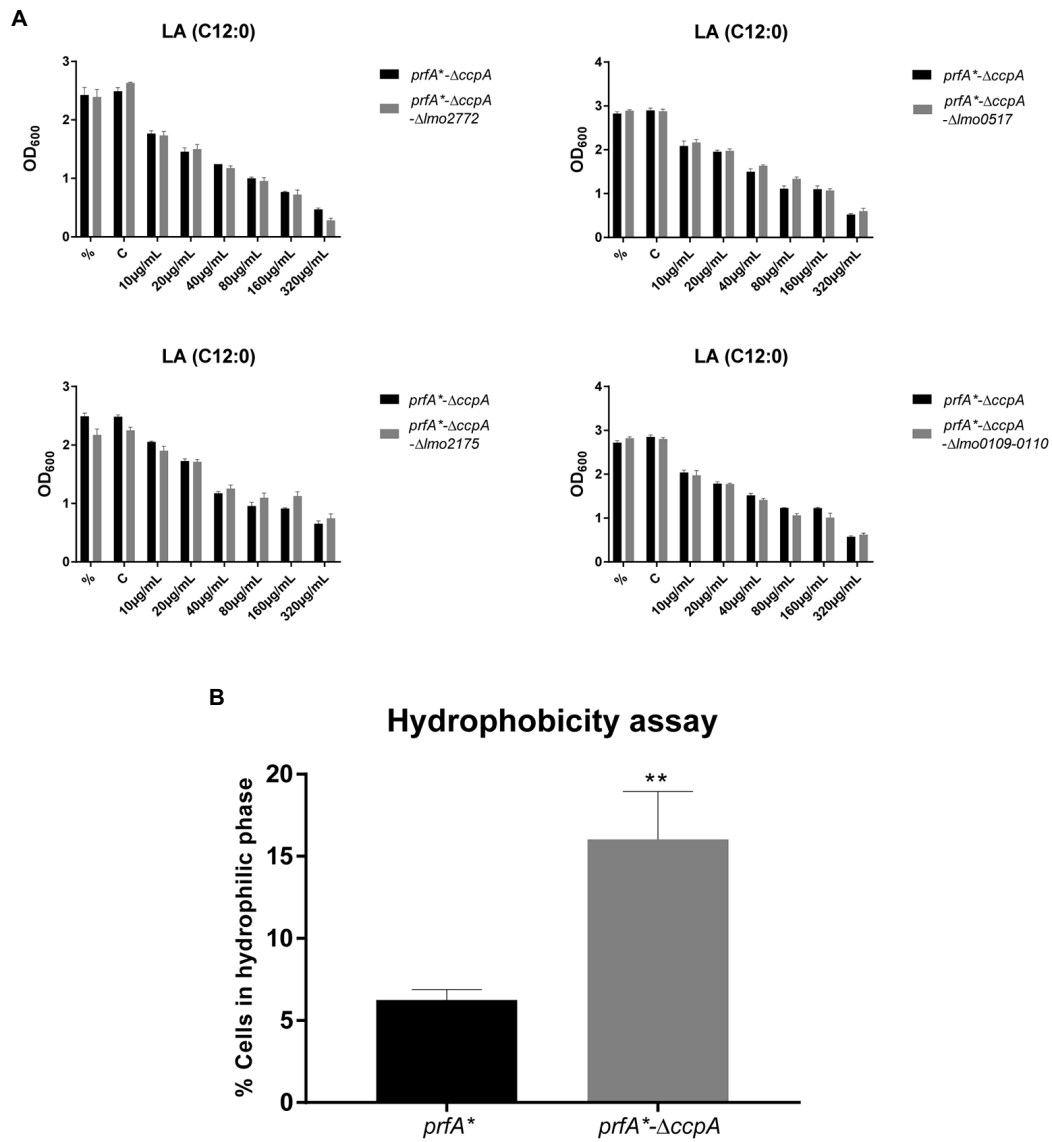
Deletion of *ccpA* results in a more hydrophilic surface. **(A)** Growth experiments for knock-out mutants with increasing concentrations of LA. ON cultures were diluted and stressed with various increasing concentrations of LA. As controls, one sample was left untreated (%) and another was stressed with a corresponding concentration of vehicle (C). Growth was measured after 20 h of growth. Results are the average of three independent experiments. **(B)** Hydrophobicity assay for prfA* and prfA*-∆*ccpA*. Bacteria were washed and diluted in 1x PBS, followed by incubation with *n*-hexadecane to measure the bacterial adhesion. OD_600_ was measured for the water phase before and after incubation with *n*-hexadecane. Data are presented as percentage of cells that stayed in the water phase, based on the OD_600_ measurements. Results are the average of three independent experiments. Statistical analysis was performed using two-tailed t-test. Only differences with at least 95% confidence were reported as statistically significant (^**^p < 0.01).

To investigate in more detail if some of the CcpA-regulated genes contribute to FFA tolerance, we focused on genes significantly upregulated by at least 4-fold in ∆*ccpA* relative to the parental strain, both under regular growth conditions and upon LA exposure (Supplementary Table S6). Two of the three most upregulated genes, chosen for mutational analysis, encode a PTS system component (*lmo2772*) and a hypothetical protein (*lmo0517*), respectively. Strikingly, these genes were upregulated more than 100-fold in the absence of CcpA (Supplementary Table S6). When going through the list of genes highly upregulated in the absence of CcpA, we furthermore noticed that the *lmo0109-lmo0110* operon is predicted to encode a transcriptional regulator and a protein with esterase/lipase function, respectively, suggesting that this operon could play a role in the response of *L. monocytogenes* to FFAs (Supplementary Table S6). Additionally, we observed that *lmo2175* (*fabG*) encodes a 3-ketoacyl-(acyl carrier protein) reductase, which catalyzes the first of two reduction steps in the elongation cycle of fatty acid synthesis (Supplementary Table S6). Knock-out mutants of *lmo2772*, *lmo0517*, the *lmo0109-lmo0110* operon, and *lmo2175* were therefore constructed in the FFA-tolerant ∆*ccpA* background, to examine if deletion of any of these genes would restore FFA sensitivity. Growth upon exposure to increasing concentrations of LA was studied for the various mutants (Figure 5A). The data revealed that deletion of the selected genes and operon, individually, does not restore FFA sensitivity in the prfA*-∆*ccpA* strain, indicating that upregulation of these CcpA-regulated genes does not confer FFA tolerance on its own. Instead, FFA tolerance might be due to altered CcpA regulation of other genes, which have not been tested in this study.

### Deletion of *ccpA* Results in a More Hydrophilic Bacterial Surface

It is well known that CcpA regulates multiple PTS systems located in the bacterial cell envelope (Schumacher et al., 2004). We therefore speculated if deletion of *ccpA* could affect the polarity of the bacterial surface. To test this idea, we compared the surface hydrophobicity of the ∆*ccpA* mutant and its parental strain by studying the microbial adhesion to *n*-hexadecane using the hydrocarbon test (Figure 5B). The ∆*ccpA* strain has an increased percentage of cells staying in the hydrophilic phase compared to its parental strain, indicating that deletion of *ccpA* confers a more hydrophilic bacterial surface. The change in surface polarity upon deletion of *ccpA* could very well be related to its FFA-tolerant phenotype. Indeed, lack of CcpA most likely increases FFA tolerance by promoting repulsion of antimicrobial FFAs, due to a more hydrophilic bacterial surface.

### CcpA Plays a Role in the Response to Acid Stress, Ampicillin, and Gentamicin

**Figure 6:**
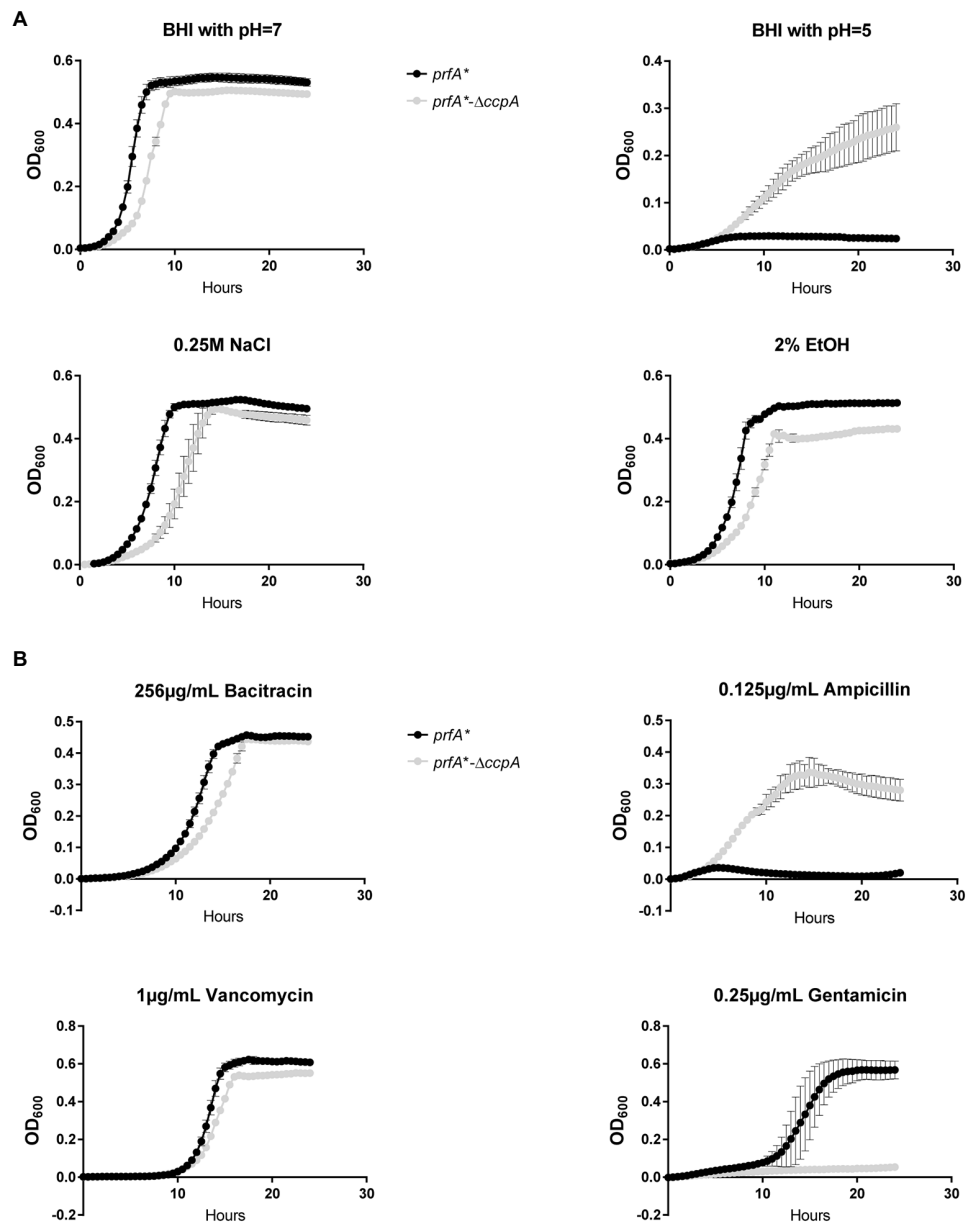
Growth of the ∆*ccpA* mutant and parental strains upon different stress conditions. **(A)** Growth upon exposure to acid, salt, or ethanol stress. ON cultures were diluted to OD_600_ = 0.005 in 96 well plates with corresponding stress conditions. Plates were incubated for 24 h at 37°C and orbitally shaken for 15 s every 30 min. Growth was measured regularly. Results are the average of three independent experiments. **(B)** Growth upon addition of different antibiotics. ON cultures were diluted to OD_600_ = 0.005 in 96-well plates with corresponding stress conditions. Plates were incubated for 24 h at 37°C and orbitally shaken for 15 s every 30 min. Growth was measured regularly. The results are the average of three independent experiments.

The experiments presented in Figure 3A clearly showed that CcpA confers sensitivity to the antimicrobial activity of LA and PA, which are known to target the bacterial membrane (Desbois and Smith, 2010). In line with this, we found that FFA tolerance of the ∆*ccpA* strain is most likely caused by changes in surface polarity (Figure 5B). These findings prompted us to investigate if CcpA plays a role in the response of *L. monocytogenes* to other stress conditions. The prfA*-∆*ccpA* and prfA* strains were subjected to growth in the presence of 2% ethanol, osmotic stress (0.25 M NaCl), acid stress (pH = 5), or antibiotics (bacitracin, ampicillin, vancomycin, gentamicin; Figure 6). Curiously, the *ccpA* deletion mutant was clearly more tolerant to low pH and ampicillin, but more sensitive to gentamicin, compared to the parental strain (Figure 6). These findings suggest that CcpA plays a role in the response of *L. monocytogenes* to acid stress conditions, as well as antibiotics used for treating listeriosis: ampicillin and gentamicin (Temple and Nahata, 2000).

## Discussion

The PrfA regulator plays a key role in the pathogenicity of *L. monocytogenes* and is essential for production of virulence factors promoting the intracellular lifestyle of this pathogen. Various signals from the environment affect the level or activity of PrfA (Scortti et al., 2007; Johansson and Freitag, 2019). The transcription of *prfA* is positively controlled by the general stress sigma factor Sigma B and the global metabolic regulator CodY, whereas translation of *prfA* mRNA is affected by temperature and two *trans*-acting S-adenosyl methionine responsive riboswitches, SreA and SreB (Johansson and Freitag, 2019; Tiensuu et al., 2019). At the post-translational level, PrfA is positively controlled by GSH which binds directly to PrfA, whereas inhibitory peptides prevent binding of GSH to PrfA (Reniere et al., 2015; Hall et al., 2016; Krypotou et al., 2019). Readily utilizable carbohydrates, such as glucose, fructose, and cellobiose, have long been known to inhibit the activity of PrfA, but the exact mechanism remains to be revealed (Milenbachs et al., 1997). Although the PrfA inhibitory carbohydrates are taken up by PTS systems, CcpA is not required for carbon source regulation of virulence genes in *L. monocytogenes* (Behari and Youngman, 1998; Deutscher et al., 2005; Herro et al., 2005; Mertins et al., 2007).

We used a prfA* strain to investigate the response of *L. monocytogenes* to antimicrobial and PrfA inhibitory FFAs. PrfA* variants are locked in a constitutively active conformation and stimulate transcription of virulence genes in BHI broth culture, where wild-type PrfA is not active (Ermolaeva et al., 2004). Importantly, PrfA* does not respond to inhibition by readily metabolizable carbohydrates (Ripio et al., 1997a) or inhibitory peptides (Krypotou et al., 2019). However, specific FFAs inhibit PrfA-dependent transcription of virulence genes in prfA* by preventing PrfA* from binding to DNA (Dos Santos et al., 2020). The transcriptome analysis of prfA* confirmed that PrfA-regulated virulence genes were indeed repressed following exposure to subinhibitory concentrations of LA (Figure 4A). Notably, less than 5 genes were differentially expressed in LA-treated cells relative to untreated cells, demonstrating that repression of virulence genes is the most prominent outcome following exposure of prfA* to LA.

At higher concentrations, LA inhibits the growth of *L. monocytogenes*, and PrfA is known to affect the sensitivity against the antimicrobial activity of LA (Sternkopf Lillebæk et al., 2017). These findings prompted us to investigate if the antimicrobial activity of LA could be linked to its PrfA inhibitory activity. We found that a frameshift mutation in *ccpA* promoted tolerance of *L. monocytogenes* to LA as well as PA, and deletion of *ccpA* generated a similar phenotype. As expected, CcpA was dispensable for PrfA-dependent activation of virulence genes in L. monocytogenes prfA* under normal growth conditions (Figure 3B). Importantly, all FFA-tolerant mutants responded well to FFA-mediated inhibition of virulence gene expression, indicating that virulence inhibitory signaling by FFAs is not linked to its antimicrobial actions. These results are well in line with recent data demonstrating that two non-antimicrobial FFAs, myristic acid (C14:0) and oleic acid (C18:1), were capable of downregulating PrfA-dependent activities, showing that antimicrobial activity is not compulsory for the PrfA inhibitory ability of an FFA (Dos Santos et al., 2020). Altogether, these findings support that exposure of *L. monocytogenes* to LA generates two separate responses: one relating to the antimicrobial activity of the FFA, and another leading to inhibition of PrfA activity. Notably, inhibition of PrfA activity may be expected to further sensitize *L. monocytogenes* to the antimicrobial actions of FFAs (Sternkopf Lillebæk et al., 2017). Future studies should aim to uncover the specific role of PrfA in mediating sensitivity to antimicrobial FFAs.

Our data clearly demonstrate that CcpA confers sensitivity toward the antimicrobial response of the FFAs, as deletion of *ccpA* caused FFA tolerance (Figure 3A). Our transcriptome analysis revealed that the FFA-tolerant phenotype, which is observed for ∆*ccpA*, does not seem to be induced upon LA exposure, but appears to be an inherent phenotype of the ∆*ccpA* mutant. Based on these observations, we hypothesized that genes underlying the FFA-tolerant phenotype are regulated by CcpA both during regular growth and upon LA exposure. To analyze this further, we deleted two genes, that are highly upregulated in the absence of CcpA (*lmo2772* and *lmo0517*), and three genes of specific interest based on their predicted protein function (*lmo2175* and *lmo0109-0110*). However, deletion of these genes did not restore FFA sensitivity in a ∆*ccpA* background (Figure 5A), suggesting that the FFA-tolerant phenotype must be based on other regulatory activities of CcpA. In Gram-positive bacteria, several mechanisms have been suggested to confer resistance to antimicrobial FFAs. In *Staphylococcus aureus*, mutant variants selected for their ability to grow in the presence of the antimicrobial FFA linoleic acid (LNA, C18:2), resulted in the identification of the fatty acid efflux pump FarE (Alnaseri et al., 2015). The LNA-tolerant mutant carried a substitution in the transcription regulator FarR, which is divergently transcribed from *farE*. The expression of *farE* is highly induced in the presence of LNA and arachidonic acid (AA, C20:4), and accordingly, FarE mediates resistance to LNA and AA, but not PA (Alnaseri et al., 2015). Notably, another efflux pump, encoded by the PA-inducible *tet38* gene, has been reported to promote resistance to PA in *S. aureus* (Truong-Bolduc et al., 2013). These studies demonstrate that FFA-tolerant phenotypes may be attributable to FFA detoxification with efflux pumps. In addition, alterations of the bacterial surface may shield the bacterium against antimicrobial FFAs. For instance, the surface protein IsdA increases the surface hydrophilicity of *S. aureus*, thereby precluding the binding of antimicrobial FFAs to the bacterium (Clarke et al., 2007). Furthermore, wall teichoic acids are known to protect *S. aureus* against antimicrobial FFAs, by repulsing FFAs from the bacterial surface (Kohler et al., 2009). Since CcpA regulates multiple genes encoding transport proteins located in the cell envelope of *L. monocytogenes*, we investigated if lack of CcpA affects the surface polarity. Our data revealed that deletion of *ccpA* results in a more hydrophilic surface of *L. monocytogenes* (Figure 5B), as observed for IsdA and wall teichoic acids in *S. aureus* (Clarke et al., 2007; Kohler et al., 2009). Based on these findings, we propose that the regulatory changes occurring by deletion of *ccpA* lead to a decrease in surface hydrophobicity, which results in a more FFA-tolerant phenotype. The specific CcpA-regulated gene(s) conferring the FFA-tolerant phenotype waits to be identified. Notably, deletion of *ccpA* not only resulted in tolerance toward antimicrobial FFAs, but also acid stress and the cell wall acting antibiotic ampicillin (Figure 6). Additionally, sensitivity was obtained toward gentamicin upon deletion of *ccpA*; an antibiotic acting to inhibit bacterial translation, which has commonly been used together with ampicillin to treat listeriosis. Together these data show that CcpA not only is involved in the response to antimicrobial FFAs; apparently, this regulator plays a broader role in the response toward multiple stress conditions encountered by *L. monocytogenes* as both a saprophyte and pathogen. Future studies should aim to reveal the regulatory role of CcpA, as well as PrfA, in responding to such conditions.

We previously suggested that FFAs acting to inhibit PrfA-dependent expression of virulence genes could be future candidates for novel anti-virulence therapies against *L. monocytogenes* (Kallipolitis, 2017; Sternkopf Lillebæk et al., 2017; Dos Santos et al., 2020). In addition to their PrfA inhibitory effect, a subset of FFAs seem particularly attractive, because they exert a dual inhibitory effect on *L. monocytogenes*: at lower concentrations, they act as signaling compounds to inhibit virulence gene expression, whereas at higher concentrations, they prevent bacterial growth. However, as for most growth inhibitory compounds, bacteria are likely to develop resistance against antimicrobial FFAs. Indeed, LA-tolerant mutant variants were readily isolated in the present study, but most importantly, the LA-tolerant strains were still susceptible to the PrfA inhibitory activity of LA. Altogether, these findings support that FFAs having a dual inhibitory effect in this pathogen may be useful candidates in future therapies against *L. monocytogenes*.

## Data Availability Statement

The datasets presented in this study can be found in online repositories. The names of the repository/repositories and accession number(s) can be found at: https://www.ncbi.nlm.nih.gov/, PRJNA815746 and PRJNA815751.

## Author Contributions

RT and BK contributed to conception and design of the study. RT, KJ, PS, and EL contributed to the experimental work. RT, MJ, MS, and MK contributed to the sequencing analysis. RT and BK wrote the first draft of the manuscript. All authors contributed to manuscript revision, read, and approved the submitted version.

## Funding

This work was supported by the Novo Nordisk Foundation (grant number NNF17OC0028528).

## Conflict of Interest

The authors declare that the research was conducted in the absence of any commercial or financial relationships that could be construed as a potential conflict of interest.

## Publisher’s Note

All claims expressed in this article are solely those of the authors and do not necessarily represent those of their affiliated organizations, or those of the publisher, the editors and the reviewers. Any product that may be evaluated in this article, or claim that may be made by its manufacturer, is not guaranteed or endorsed by the publisher.

## References

[ref1] Alnaseri H. Arsic B. Schneider J. E. Kaiser J. C. Scinocca Z. C. Heinrichs D. E.. (2015). Inducible expression of a resistance-nodulation-division-type efflux pump in *Staphylococcus aureus* provides resistance to linoleic and arachidonic acids. J. Bacteriol. 197, 1893–1905. doi: 10.1128/JB.02607-14, PMID: 25802299 PMC4420908

[ref2] Bécavin C. Bouchier C. Lechat P. Archambaud C. Creno S. Gouin E.. (2014). Comparison of widely used *Listeria monocytogenes* strains EGD, 10403S, and EGD-e highlights genomic variations underlying differences in pathogenicity. mBio 5, e00969–e00914. 24667708 10.1128/mBio.00969-14 PMC3977354

[ref3] Behari J. Youngman P. (1998). A homolog of CcpA mediates catabolite control in *listeria monocytogenes* but not carbon source regulation of virulence genes. J. Bacteriol. 180, 6316–6324. doi: 10.1128/JB.180.23.6316-6324.1998, PMID: 9829942 PMC107718

[ref4] Böckmann R. Dickneite C. Middendorf B. Goebel W. Sokolovic Z. (1996). Specific binding of the *Listeria monocytogenes* transcriptional regulator PrfA to target sequences requires additional factor(s) and is influenced by iron. Mol. Microbiol. 22, 643–653. doi: 10.1046/j.1365-2958.1996.d01-1722.x, PMID: 8951812

[ref6] Chaptal V. Gueguen-Chaignon V. Poncet S. Lecampion C. Meyer P. Deutscher J.. (2006). Structural analysis of *B. subtilis* CcpA effector binding site. Proteins 64, 814–816. doi: 10.1002/prot.21001, PMID: 16755587

[ref7] Christiansen J. K. Larsen M. H. Ingmer H. Søgaard-Andersen L. Kallipolitis B. H. (2004). The RNA-binding protein Hfq of *Listeria monocytogenes*: role in stress tolerance and virulence. J. Bacteriol. 186, 3355–3362. doi: 10.1128/JB.186.11.3355-3362.2004, PMID: 15150220 PMC415768

[ref8] Clarke S. R. Mohamed R. Bian L. Routh A. F. Kokai-Kun J. F. Mond J. J.. (2007). The *Staphylococcus aureus* surface protein IsdA mediates resistance to innate defenses of human skin. Cell Host Microbe 1, 199–212. doi: 10.1016/j.chom.2007.04.005, PMID: 18005699

[ref9] de las Heras A. Cain R. J. Bielecka M. K. Vázquez-Boland J. A. (2011). Regulation of *listeria* virulence: PrfA master and commander. Curr. Opin. Microbiol. 14, 118–127. doi: 10.1016/j.mib.2011.01.005 21388862

[ref10] Deatherage D. E. Barrick J. E. (2014). Identification of mutations in laboratory-evolved microbes from next-generation sequencing data using breseq. Methods Mol. Biol. 1151, 165–188. doi: 10.1007/978-1-4939-0554-6_12, PMID: 24838886 PMC4239701

[ref11] Desbois A. P. Smith V. J. (2010). Antibacterial free fatty acids: activities, mechanisms of action and biotechnological potential. Appl. Microbiol. Biotechnol. 85, 1629–1642. doi: 10.1007/s00253-009-2355-3, PMID: 19956944

[ref12] Deutscher J. (2008). The mechanisms of carbon catabolite repression in bacteria. Curr. Opin. Microbiol. 11, 87–93. doi: 10.1016/j.mib.2008.02.007 18359269

[ref13] Deutscher J. Herro R. Bourand A. Mijakovic I. Poncet S. (2005). P-Ser-HPr: A link between carbon metabolism and the virulence of some pathogenic bacteria. Biochim. Biophys. Acta 1754, 118–125. doi: 10.1016/j.bbapap.2005.07.029, PMID: 16182622

[ref14] Dos Santos P. T. Thomasen R. S. S. Green M. S. Færgeman N. J. Kallipolitis B. H. (2020). Free fatty acids interfere with the DNA binding activity of the virulence regulator PrfA of *Listeria monocytogenes*. J. Bacteriol. 202, e00156–e00120. doi: 10.1128/JB.00156-20 32393522 PMC7348547

[ref15] Eiting M. Hageluken G. Schubert W. D. Heinz D. W. (2005). The mutation G145S in PrfA, a key virulence regulator of *Listeria monocytogenes*, increases DNA-binding affinity by stabilizing the HTH motif. Mol. Microbiol. 56, 433–446. doi: 10.1111/j.1365-2958.2005.04561.x, PMID: 15813735

[ref16] Ermolaeva S. Novella S. Vega Y. Ripio M. T. Scortti M. Vázquez-Boland J. A. (2004). Negative control of *Listeria monocytogenes* virulence genes by a diffusible autorepressor. Mol. Microbiol. 52, 601–611. doi: 10.1111/j.1365-2958.2004.04003.x, PMID: 15066044

[ref17] Freitag N. E. Port G. C. Miner M. D. (2009). *Listeria monocytogenes* – from saprophyte to intracellular pathogen. Nat. Rev. Microbiol. 7, 623–628. doi: 10.1038/nrmicro2171, PMID: 19648949 PMC2813567

[ref18] Galinier A. Haiech J. Kilhoffer M. C. Jaquinod M. Stülke J. Deutscher J.. (1997). The *Bacillus subtilis* crh gene encodes a HPr-like protein involved in carbon catabolite repression. Proc. Natl. Acad. Sci. U. S. A. 94, 8439–8444. doi: 10.1073/pnas.94.16.8439, PMID: 9237995 PMC22949

[ref19] Hall M. Grundstrom C. Begum A. Lindberg M. J. Sauer U. H. Almqvist F.. (2016). Structural basis for glutathione-mediated activation of the virulence regulatory protein PrfA in *Listeria*. Proc. Natl. Acad. Sci. U. S. A. 113, 14733–14738. doi: 10.1073/pnas.1614028114, PMID: 27930316 PMC5187702

[ref20] Herro R. Poncet S. Cossart P. Buchrieser C. Gouin E. Glaser P.. (2005). How seryl-phosphorylated HPr inhibits PrfA, a transcription activator of *Listeria monocytogenes* virulence genes. J. Mol. Microbiol. Biotechnol. 9, 224–234. doi: 10.1159/000089650 16415595

[ref21] Johansson J. Freitag N. E. (2019). Regulation of *Listeria monocytogenes* virulence. Microbiol. Spectr. 7, 1–20. doi: 10.1128/microbiolspec.GPP3-0064-2019 PMC10957223 31441398

[ref22] Jones B. E. Dossonnet V. Küster E. Hillen W. Deutscher J. Klevit R. E. (1997). Binding of the catabolite repressor protein CcpA to its DNA target is regulated by phosphorylation of its corepressor HPr. J. Biol. Chem. 272, 26530–26535. doi: 10.1074/jbc.272.42.26530, PMID: 9334231

[ref23] Kallipolitis B. H. (2017). How can naturally occurring fatty acids neutralize *Listeria*? Future Microbiol. 12, 1239–1241. doi: 10.2217/fmb-2017-0176 28975811

[ref24] Kohler T. Weidenmaier C. Peschel A. (2009). Wall teichoic acid protects *Staphylococcus aureus* against antimicrobial fatty acids from human skin. J. Bacteriol. 191, 4482–4484. doi: 10.1128/JB.00221-09, PMID: 19429623 PMC2698495

[ref25] Krypotou E. Scortti M. Grundström C. Oelker M. Luisi B. F. Sauer-Eriksson A. E.. (2019). Control of bacterial virulence through the peptide signature of the habitat. Cell Rep. 26, 1815.e5–1827.e5. doi: 10.1016/j.celrep.2019.01.073, PMID: 30759392 PMC6389498

[ref26] Langmead B. Salzberg S. L. (2012). Fast gapped-read alignment with bowtie 2. Nat. Methods 9, 357–359. doi: 10.1038/nmeth.1923, PMID: 22388286 PMC3322381

[ref27] Larsen M. H. Kallipolitis B. H. Christiansen J. K. Olsen J. E. Ingmer H. (2006). The response regulator ResD modulates virulence gene expression in response to carbohydrates in *Listeria monocytogenes*. Mol. Microbiol. 61, 1622–1635. doi: 10.1111/j.1365-2958.2006.05328.x, PMID: 16968229

[ref28] Mertins S. Joseph B. Goetz M. Ecke R. Seidel G. Sprehe M.. (2007). Interference of components of the phosphoenolpyruvate phosphotransferase system with the central virulence gene regulator PrfA of *Listeria monocytogenes*. J. Bacteriol. 189, 473–490. doi: 10.1128/JB.00972-06, PMID: 17085572 PMC1797385

[ref29] Milenbachs A. A. Brown D. P. Moors M. Youngman P. (1997). Carbon-source regulation of virulence gene expression in *Listeria monocytogenes*. Mol. Microbiol. 23, 1075–1085. doi: 10.1046/j.1365-2958.1997.2711634.x, PMID: 9076743

[ref30] Mollerup M. S. Ross J. A. Helfer A. C. Meistrup K. Romby P. Kallipolitis B. H. (2016). Two novel members of the LhrC family of small RNAs in *Listeria monocytogenes* with overlapping regulatory functions but distinctive expression profiles. RNA Biol. 13, 895–915. doi: 10.1080/15476286.2016.1208332, PMID: 27400116 PMC5013991

[ref31] Nielsen J. S. Larsen M. H. Lillebæk E. M. Bergholz T. M. Christiansen M. H. Boor K. J.. (2011). A small RNA controls expression of the chitinase ChiA in *Listeria monocytogenes*. PLoS One 6:e19019. doi: 10.1371/journal.pone.0019019, PMID: 21533114 PMC3078929

[ref32] Nielsen J. S. Lei L. K. Ebersbach T. Olsen A. S. Klitgaard J. K. Valentin-Hansen P.. (2010). Defining a role for Hfq in gram-positive bacteria: evidence for Hfq-dependent antisense regulation in *Listeria monocytogenes*. Nucleic Acids Res. 38, 907–919. doi: 10.1093/nar/gkp1081, PMID: 19942685 PMC2817478

[ref33] Petrone G. Conte M. P. Longhi C. di Santo S. Superti F. Ammendolia M. G.. (1998). Natural milk fatty acids affect survival and invasiveness of *listeria monocytogenes*. Lett. Appl. Microbiol. 27, 362–368. doi: 10.1046/j.1472-765X.1998.00441.x, PMID: 9871355

[ref34] Reniere M. L. Whiteley A. T. Hamilton K. L. John S. M. Lauer P. Brennan R. G.. (2015). Glutathione activates virulence gene expression of an intracellular pathogen. Nature 517, 170–173. doi: 10.1038/nature14029, PMID: 25567281 PMC4305340

[ref35] Ripio M. T. Brehm K. Lara M. Suarez M. Vázquez-Boland J. A. (1997a). Glucose-1-phosphate utilization by *Listeria monocytogenes* is PrfA dependent and coordinately expressed with virulence factors. J. Bacteriol. 179, 7174–7180. doi: 10.1128/jb.179.22.7174-7180.1997, PMID: 9371468 PMC179662

[ref36] Ripio M. T. Dominguez-Bernal G. Lara M. Suarez M. Vázquez-Boland J. A. (1997b). A Gly145Ser substitution in the transcriptional activator PrfA causes constitutive overexpression of virulence factors in *listeria monocytogenes*. J. Bacteriol. 179, 1533–1540. doi: 10.1128/jb.179.5.1533-1540.1997, PMID: 9045810 PMC178863

[ref37] Schäferkordt S. Chakraborty T. (1995). Vector plasmid for insertional mutagenesis and directional cloning in *Listeria* spp. BioTechniques 19, 720–725. 8588903

[ref38] Schumacher M. A. Allen G. S. Diel M. Seidel G. Hillen W. Brennan R. G. (2004). Structural basis for allosteric control of the transcription regulator CcpA by the phosphoprotein HPr-Ser46-P. Cell 118, 731–741. doi: 10.1016/j.cell.2004.08.027, PMID: 15369672

[ref39] Scortti M. Monzó H. J. Lacharme-Lora L. Lewis D. A. Vázquez-Boland J. A. (2007). The PrfA virulence regulon. Microbes Infect. 9, 1196–1207. doi: 10.1016/j.micinf.2007.05.007, PMID: 17764998

[ref40] Sternkopf Lillebæk E. M. Lambert Nielsen S. Scheel Thomasen R. Færgeman N. J. Kallipolitis B. H. (2017). Antimicrobial medium- and long-chain free fatty acids prevent PrfA-dependent activation of virulence genes in *Listeria monocytogenes*. Res. Microbiol. 168, 547–557. doi: 10.1016/j.resmic.2017.03.002, PMID: 28344104

[ref41] Temple M. E. Nahata M. C. (2000). Treatment of listeriosis. Ann. Pharmacother. 34, 656–661. doi: 10.1345/aph.19315 10852095

[ref42] Tiensuu T. Guerreiro D. N. Oliveira A. H. O'Byrne C. Johansson J. (2019). Flick of a switch: regulatory mechanisms allowing *Listeria monocytogenes* to transition from a saprophyte to a killer. Microbiology 165, 819–833. doi: 10.1099/mic.0.000808, PMID: 31107205

[ref43] Truong-Bolduc Q. C. Villet R. A. Estabrooks Z. A. Hooper D. C. (2013). Native efflux pumps contribute resistance to antimicrobials of skin and the ability of *Staphylococcus aureus* to colonize skin. J. Infect. Dis. 209, 1485–1493. doi: 10.1093/infdis/jit660 24280365 PMC3982850

[ref44] Xayarath B. Freitag N. E. (2012). Optimizing the balance between host and environmental survival skills: lessons learned from *Listeria monocytogenes*. Future Microbiol. 7, 839–852. doi: 10.2217/fmb.12.57, PMID: 22827306 PMC3479242

