# Supplementary material for: The Global Regulator CcpA of Listeria monocytogenes Confers Sensitivity to Antimicrobial Fatty Acids
Source: Front Microbiol. 2022 May 3;13:895942. doi: 10.3389/fmicb.2022.895942 (PMC9113694; doi:10.3389/fmicb.2022.895942)
Supplement: Supplementary file 1 [file Data_Sheet_1.PDF]

Supplementary material

The global regulator CcpA of *Listeria monocytogenes* confers sensitivity to antimicrobial fatty acids

Rikke S. S. Thomasen[1], Magnus Ganer Jespersen[1,2], Katrine Jørgensen[1], Patricia T. dos Santos[1,3], Eva Maria Sternkopf Lillebæk[1], Marianne N. Skov[4], Michael Kemp[4,5] and Birgitte H. Kallipolitis[1*]

[1]Department of Biochemistry and Molecular Biology, University of Southern Denmark, Odense, Denmark.

[2]Department of Microbiology and Immunology, The Peter Doherty Institute for Infection and Immunity, University of Melbourne, Melbourne, VIC, Australia.

[3]National Food Institute, Technical University of Denmark, Kgs. Lyngby, Denmark.

[4]Department of Clinical Microbiology, Odense University Hospital and Research Unit of Clinical Microbiology, University of Southern Denmark, Odense, Denmark.

[5]The Regional Department of Clinical Microbiology, Region Zealand, Zealand University Hospital, Koege, Denmark.

***Correspondence:** Birgitte H. Kallipolitis, bhk@bmb.sdu.dk

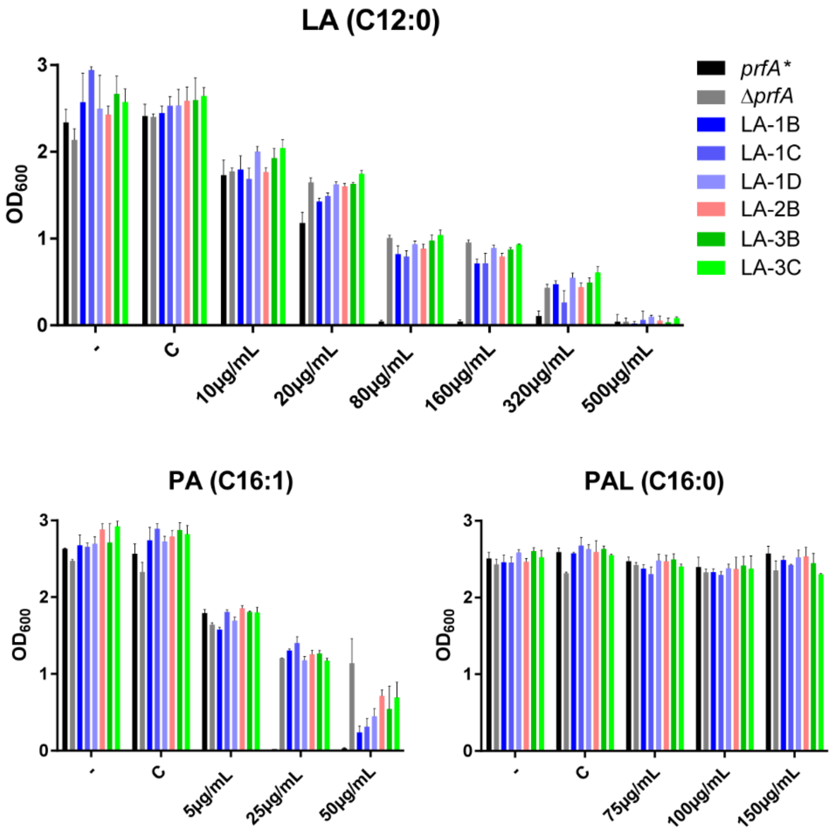

Suppl. Figure S1: Growth of isolated LA-tolerant strains in presence of increasing concentrations of FFAs. ON-cultures of *prfA**, Δ*prfA* and the LA-tolerant strains (LA-1B, LA-1C, LA-1D, LA-2B, LA-3B, LA-3C; see Table 1 for details) were diluted and incubated with increasing concentrations of lauric acid (LA), palmitoleic acid (PA) and palmitic acid (PAL). Growth was measured after 20 h. Results are the average of at least three independent experiments.

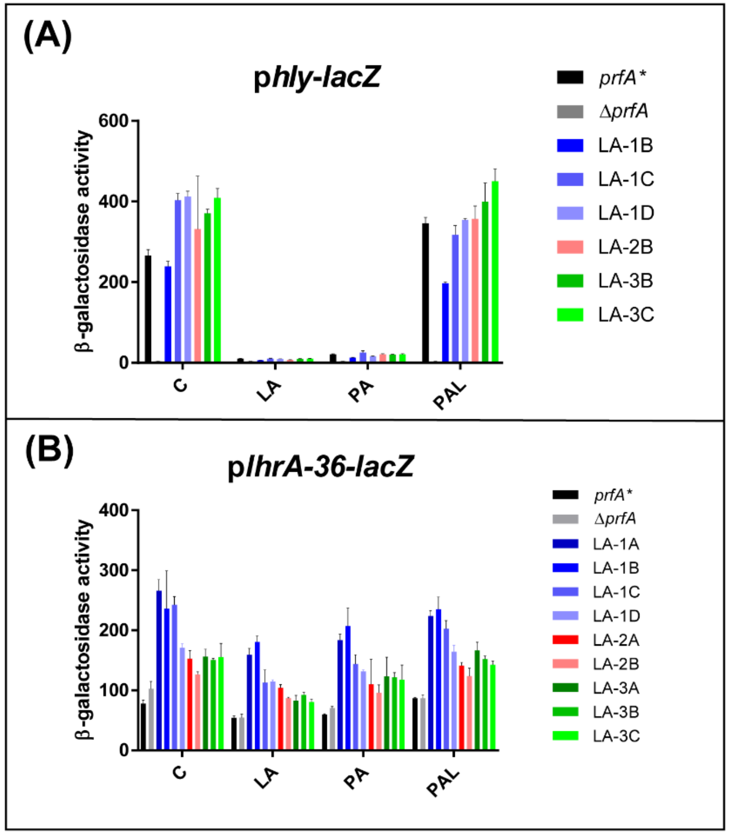

Suppl. Figure S2: LA-tolerant strains retain sensitivity towards the PrfA-inhibitory effect of FFAs. ON-cultures of *prfA**, Δ*prfA* and the LA tolerant strains (LA-1A, LA-1B, LA-1C, LA-1D, LA-2A, LA-2B, LA-3A, LA-3B, LA-3C; see Table 1 for details) transformed with p*hly*-*lacZ*, containing a transcriptional fusion of the *hly* promoter to the *lacZ* gene (**A**), or p*lhrA*-36-*lacZ*, containing a transcriptional fusion of the PrfA-independent *lhrA* promoter to *lacZ* (**B**) were diluted into BHI medium. Cultures were grown to early exponential phase and stressed with either 10 µg/mL lauric acid (LA), 2 µg/mL palmitoleic acid (PA), or 150 µg/mL palmitic acid (PAL). As controls, cultures were exposed to a corresponding concentration of vehicle (C). Results are the average of three independent experiments each performed in technical duplicates.

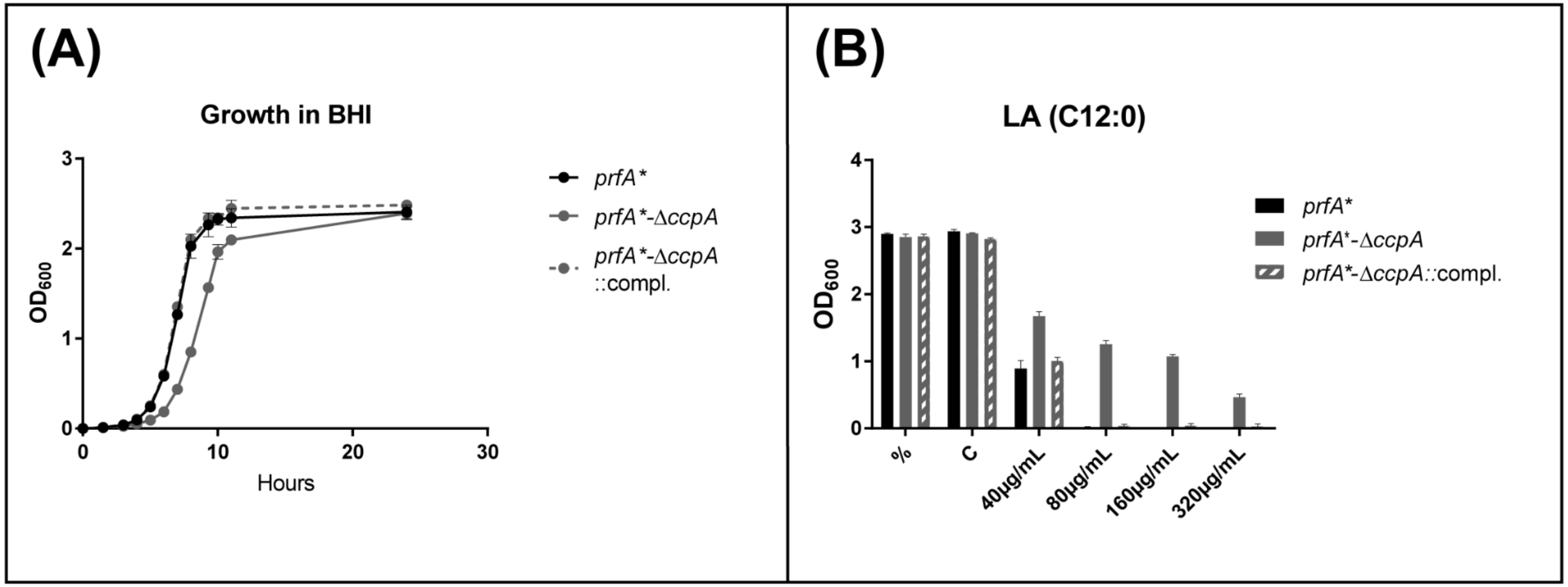

Suppl. Figure S3: Complementation of Δ*ccpA* with wild-type *ccpA*. (**A**) Growth in regular BHI. Strains *prfA**, *prfA**-Δ*ccpA* and *prfA**-Δ*ccpA::*compl were diluted to OD_{600}=0.002. Cultures were incubated under standard conditions and growth was measured regularly. (**B**) Growth upon increasing concentrations of lauric acid (LA). ON-cultures of *prfA**, *prfA**-Δ*ccpA* and *prfA**-Δ*ccpA::*compl were diluted and exposed to increasing concentrations of LA. As control, cultures were left untreated (%) or exposed to a corresponding concentration of vehicle (C). Growth was measured after 20 h. Results are the average of three independent experiments.

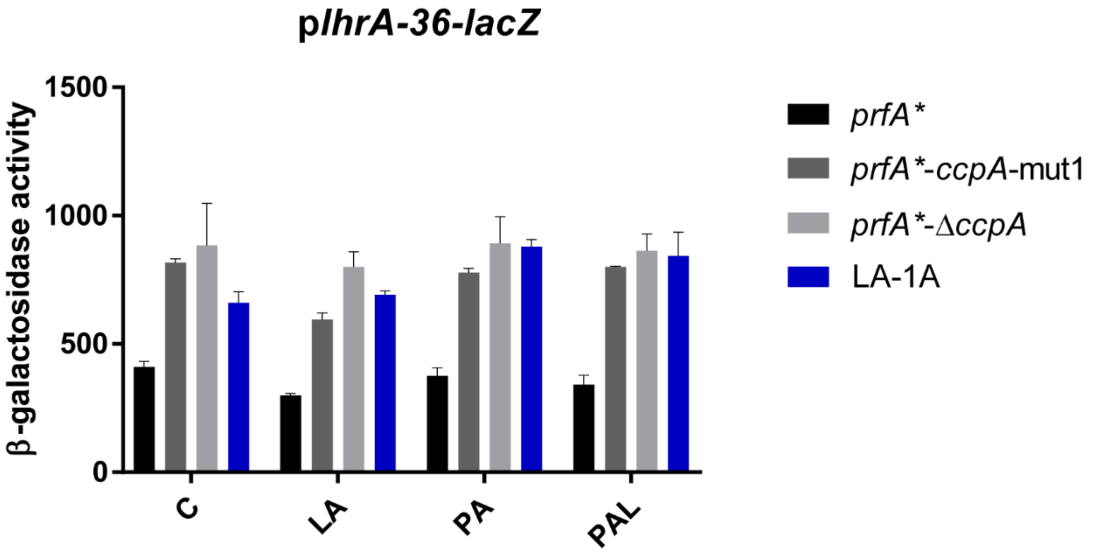

Suppl. Figure S4: FFAs do not inhibit PrfA-independent promotor activity. ON-cultures of *prfA**, *prfA*-ccpA*-mut1, *prfA**-Δ*ccpA* and LA-1A (see Table 1 for details) transformed with p*lhrA*-36-*lacZ*, a transcriptional fusion of the PrfA-independent *lhrA* promoter to *lacZ*, were diluted in BHI medium. Cultures were grown to early exponential phase and stressed with either 10 µg/mL lauric acid (LA), 2 µg/mL palmitoleic acid (PA), or 150 µg/mL palmitic acid (PAL). As controls, cultures were exposed to a corresponding concentration of vehicle (C). Results are the average of three independent experiments each performed in technical duplicates.

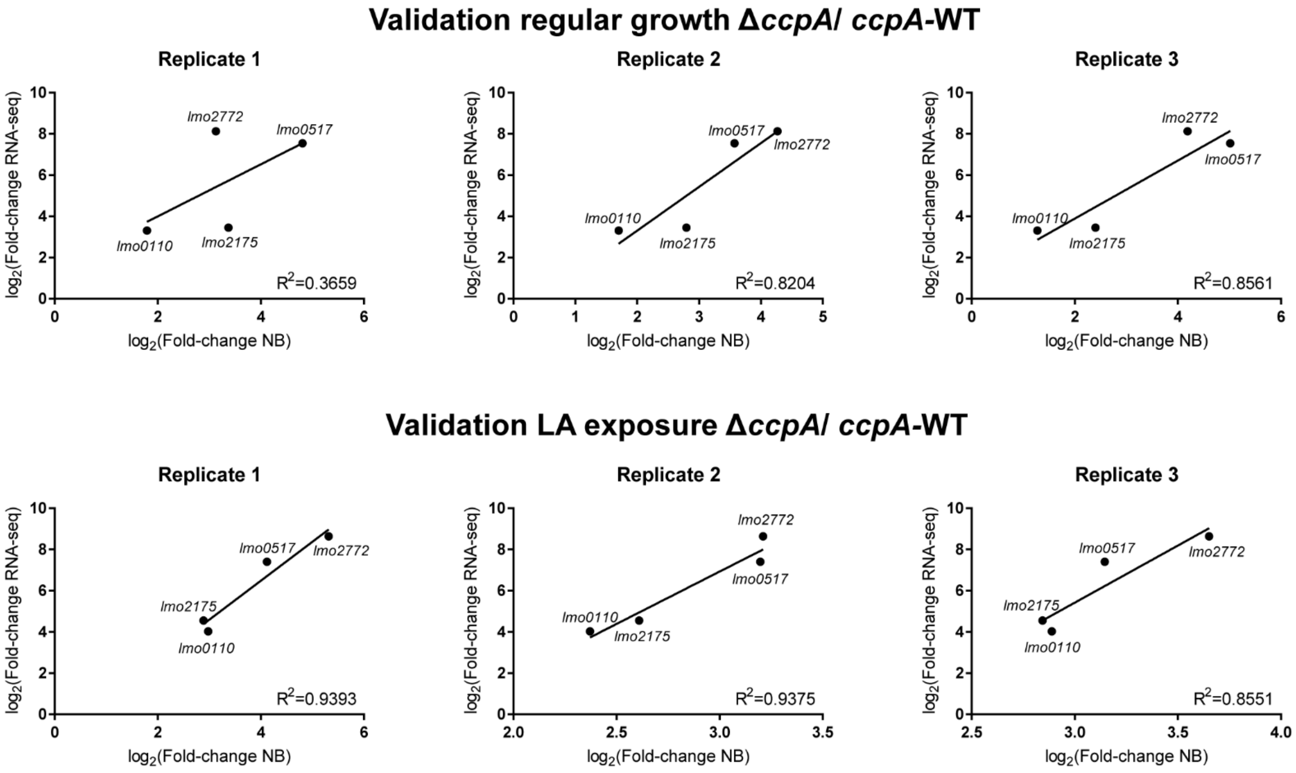

Suppl. Figure S5: Validation of RNA sequencing by northern blot analysis. The expression of the 4 studied genes *lmo0110* (from the *lmo0109-0110* operon), *lmo2175, lmo0517* and *lmo2772* were studied by northern blot analysis. Samples were taken from *prfA** and *prfA**-Δ*ccpA* cultures upon regular growth and exposure to 10 µg/mL lauric acid (LA). Northern blots were performed for regular growth and LA exposure, respectively, and probed for the four selected mRNAs and 16S mRNA (loading control). $\log_2$ to the relative level of mRNA (normalized to 16S) ($\log_2$(Fold-change NB)) were plotted against $\log_2$ to the fold change from the RNA sequencing ($\log_2$(Fold-change RNA-seq)) for each of the three independent experiments from the northern blot to compare the fold changes obtained in the RNA-seq and the northern blot analysis.

Supplementary Table S1: Doubling time (T2) for *ccpA* mutants and corresponding parental strains.

**Strain**	**T2 (minutes)**	**% Increase**
Wild-type	38.9	-
ccpA-mut1	39.8	2.3%
Δ*ccpA*	47.5	21.9%
*prfA**	42.8	-
*prfA**-*ccpA*-mut1	46.2	8%
*prfA**-Δ*ccpA*	58.2	36.1%
[1]LA-1A	46.2	8%

[1]Selected LA-tolerant strain carrying a frameshift mutation in *ccpA*. See Table 1 for details.

Supplementary Table S2: Genes ≥ 2.0-fold up-regulated and ≤ -2.0-fold down-regulated (p ≤0.05) in *Listeria monocytogenes prfA** cells exposed to 10µg/mL LA for 1hour compared to non-stressed cells.

Gene symbol[1]	Gene name	Fold change	Description[2]	Clusters of Orthologous Genes (COGs)[2]
lmo2069	*groES*	2.0	Co-chaperonin GroES	Posttranslational modification. protein turnover. chaperones
lmo0201	*plcA*	-12.3	Phosphatidylinositol-specific phospholipase c	Defense/virulence mechanisms
lmo0202	*hly*	-5.9	Listeriolysin O precursor	Defense/virulence mechanisms
lmo0204	*actA*	-5.6	Actin-assembly inducing protein precursor	Cell motility

[1] *lmo* denotes a protein coding gene.

[2] Information from Listeriomics website (https://listeriomics.pasteur.fr/Listeriomics/#bacnet.Listeria). If only Rapid Annotations using Subsystems Technology (RAST)-products of the gene were found, RAST-products are listed as Description.

Supplementary Table S3: Genes ≥ 2.0-fold up-regulated and ≤ -2.0-fold down-regulated ($p \leq 0.05$) in *Listeria monocytogenes prfA**-Δ*ccpA* cells exposed to 10µg/mL LA for 1hour compared to non-stressed cells.

Gene symbol[1]	Gene name	Fold change	Description[2]	Clusters of Orthologous Genes (COGs)[2]
lmo2125		2.7	Maltose/maltodextrin ABC transporter. substrate binding periplasmic protein MalE	Carbohydrate transport and metabolism
lmo0097		2.5	PTS system. mannose-specific IIC component	Carbohydrate transport and metabolism
lmo2126		2.3	Neopullulanase	Carbohydrate transport and metabolism
lmo0783		2.3	PTS system. mannose-specific IIB component	Carbohydrate transport and metabolism
lmo0784		2.3	PTS system. mannose-specific IIB component / PTS system. mannose-specific IIA component	Carbohydrate transport and metabolism
lmo0096		2.21	PTS system. mannose-specific IIB component / PTS system. mannose-specific IIA component	Carbohydrate transport and metabolism
lmo0201	*plcA*	-11.1	Phosphatidylinositol-specific phospholipase c	Defense/virulence mechanisms
lmo0202	*hly*	-7.1	Listeriolysin O precursor	Defense/virulence mechanisms
lmo0204	*actA*	-5.6	Actin-assembly inducing protein precursor	Cell motility
lmo0205	*plcB*	-5.0	Phospholipase C	Defense/virulence mechanisms
lmo0838	*uhpT*	-4.7	Sugar phosphate antiporter	Carbohydrate transport and metabolism
lmo0203	*mpl*	-4.4	Zinc metalloproteinase precursor	Amino acid transport and metabolism
lmo2156		-2.8	Hypothetical protein	Function unknown
lmo2439		-2.6	Hypothetical protein	Function unknown

[1] *lmo* denotes a protein coding gene.

[2] Information from Listeriomics website (https://listeriomics.pasteur.fr/Listeriomics/#bacnet.Listeria). If only Rapid Annotations using Subsystems Technology (RAST)-products of the gene was found, RAST-products are listed as Description.

Supplementary Table S4: Genes ≥ 2.0-fold up-regulated and ≤ -2.0-fold down-regulated ($p \leq 0.05$) in *Listeria monocytogenes prfA*-ΔccpA* compared to *prfA** during growth in BHI.

Gene symbol[1]	Gene name	Fold change	Description[2]	Clusters of Orthologous Genes (COGs)[2]
lmo2771		279.49	6-phospho-beta-glucosidase	Carbohydrate transport and metabolism
lmo2772		269.40	PTS system, beta-glucoside-specific IIB component / PTS system, beta-glucoside-specific IIC component / PTS system, beta-glucoside-specific IIA component	Carbohydrate transport and metabolism
lmo0517		186.85	Hypothetical, related to broad specificity phosphatases COG0406	Carbohydrate transport and metabolism
lmo1879	*cspD*	93.80	Cold shock protein CspB	Transcription
lmo0681		79.03	Flagellar biosynthesis regulator FlhF	Cell motility
lmo2787	*bvrB*	69.07	Beta-glucoside-specific phosphotransferase enzyme II ABC component	Carbohydrate transport and metabolism
lmo2798		67.76	Hydrolase, haloacid dehalogenase-like family	General function prediction only
lmo0027		62.60	PTS system, beta-glucoside-specific IIB component / PTS system, beta-glucoside-specific IIC component / PTS system, beta-glucoside-specific IIA component	Carbohydrate transport and metabolism
lmo2585		59.15	Hypothetical protein YrhD	Function unknown
lmo1731		49.10	Hypothetical protein YrhD	Function unknown
lmo2586		45.58	Formate dehydrogenase related protein	General function prediction only
lmo0024		41.67	Hypothetical protein	Carbohydrate transport and metabolism
lmo0643		40.45	Hypothetical protein	Carbohydrate transport and metabolism
lmo0025		37.08	Hypothetical protein	General function prediction only
lmo0105		35.31	Hypothetical protein	Carbohydrate transport and metabolism; General function prediction only
lmo1997		34.66	PTS system, mannose-specific IIA component	Carbohydrate transport and metabolism
lmo0697	*flgE*	34.04	Flagellar hook protein FlgE	Cell motility
lmo2584		32.58	Formate dehydrogenase accessory protein	Energy production and conversion
lmo0109		31.67	Transcriptional regulator, AraC family	Transcription
lmo0357		30.17	PTS system, fructose-specific IIA component	Carbohydrate transport and metabolism; Signal transduction mechanisms
lmo2409		29.38	Hypothetical protein	Not in COGs
lmo0023		28.73	PTS system, mannose-specific IIC component	Carbohydrate transport and metabolism

lmo2683		28.40	PTS system, cellobiose-specific IIB component	Carbohydrate transport and metabolism
lmo1999		26.22	Glucosamine—fructose-6-phosphate aminotransferase [isomerizing]	Cell wall/membrane biogenesis
lmo2773		26.19	Beta-glucoside bgl operon antiterminator, BglG family	Transcription
lmo1042		25.49	Molybdopterin biosynthesis protein MoeA	Coenzyme transport and metabolism
lmo2163		24.28	Myo-inositol 2-dehydrogenase 1	General function prediction only
lmo0181		23.59	N-Acetyl-D-glucosamine ABC transport system, sugar-binding protein	Carbohydrate transport and metabolism
lmo1043		22.92	Molybdopterin-guanine dinucleotide biosynthesis protein MobB	Coenzyme transport and metabolism
lmo1968		21.15	Creatinine amidohydrolase	General function prediction only
lmo0385		20.60	5-keto-2-deoxygluconokinase	Carbohydrate transport and metabolism
lmo0675		20.51	Flagellar motor switch protein FliN	Not in COGs
lmo1045		20.24	Molybdenum cofactor biosynthesis protein MoaD	Coenzyme transport and metabolism
lmo0685		20.20	Flagellar motor protein MotA	Cell motility
lmos25		19.77	No description	
lmo1338		19.58	Hypothetical protein	Function unknown
lmos31		19.48	No description	
lmo2666		19.29	PTS system, galactitol-specific IIB component	Carbohydrate transport and metabolism
lmo0021		19.04	PTS system, IIA component	Carbohydrate transport and metabolism
lmo1254		18.66	Trehalose-6-phosphate hydrolase	Carbohydrate transport and metabolism
lmo0877		18.57	Glucosamine-6-phosphate deaminase	arbohydrate transport and metabolism
lmo0702		17.85	Hypothetical protein	Function unknown
lmo2851		17.59	Transcriptional regulator of rhamnose utilization, AraC family	Transcription
lmo2708		17.14	PTS system, cellobiose-specific IIC component	Carbohydrate transport and metabolism
lmo0718		16.42	Hypothetical protein	Not in COGs
lmo0693		16.27	Flagellar motor switch protein	Cell motility; Intracellular trafficking and secretion
lmo2786	*bvrC*	16.04	ADP-ribosylglycohydrolase YegU	Posttranslational modification, protein turnover, chaperones
lmo0022		15.86	PTS system, mannose-specific IIB component / PTS system, mannose-specific IIA component	Carbohydrate transport and metabolism
lmos48		15.72	No description	
lmos22		15.55	No description	
lmo0323		15.15	Endonuclease/exonuclease/phosphatase family protein	General function prediction only

lmos19		14.91	No description	
lmos34		14.40	No description	
lmo0679	*flhB*	13.65	Flagellar biosynthesis protein FlhB	Cell motility; Intracellular trafficking and secretion
lmo1044		13.53	Molybdenum cofactor biosynthesis protein MoaE	Coenzyme transport and metabolism
lmo2651		13.27	PTS system, IIA component	Carbohydrate transport and metabolism; Signal transduction mechanisms
lmo0862		13.24	Trehalose-6-phosphate hydrolase	Carbohydrate transport and metabolism
lmo0776		13.22	Fructokinase	Transcription; Carbohydrate transport and metabolism
lmo0768		13.06	Sugar ABC transporter, sugar-binding protein	Carbohydrate transport and metabolism
lmo0508		12.80	PTS system, galactitol-specific IIC component	Carbohydrate transport and metabolism
lmo0543		12.72	PTS system, glucitol/sorbitol-specific IIB component and second of two IIC components	Carbohydrate transport and metabolism
lmo0859		12.65	Multiple sugar ABC transporter, substrate-binding protein	Carbohydrate transport and metabolism
lmo0682	*flgG*	12.64	Flagellar basal body rod protein FlgG	Cell motility
lmot41		12.62	No description	
lmo2099		12.41	Predicted galactitol operon regulator (Transcriptional antiterminator), BglG family / PTS system, mannitol/fructose-specific IIA component	Transcription; Carbohydrate transport and metabolism; Signal transduction mechanisms
lmo1047	*moaA*	12.16	Molybdenum cofactor biosynthesis protein A	Coenzyme transport and metabolism
lmo2801		12.00	N-acetylmannosamine-6-phosphate 2-epimerase	Carbohydrate transport and metabolism
lmo0038		11.88	Agmatine deiminase	Amino acid transport and metabolism
lmo2000		11.69	PTS system, mannose-specific IID component	Carbohydrate transport and metabolism
lmo0713	*fliF*	11.51	Flagellar MS-ring protein	Cell motility; Intracellular trafficking and secretion
lmo0151		11.33	Hypothetical protein	Not in COGs
lmo0901		11.31	PTS system, cellobiose-specific IIC component	Carbohydrate transport and metabolism
lmo0683		11.27	Chemotaxis protein methyltransferase CheR	Cell motility; Signal transduction mechanisms
lmo0033		11.24	glycosyl hydrolase, family 9	Not in COGs
lmo0868		11.18	hypothetical protein	Transcription
lmo2175	*fabG*	10.98	3-ketoacyl-(acyl-carrier-protein) reductase	Lipid transport and metabolism; Secondary metabolites biosynthesis, transport and catabolism; General function prediction only
lmo1349		10.90	Glycine dehydrogenase subunit 1	Amino acid transport and metabolism
lmo1348	*gcvT*	10.88	Glycine cleavage system aminomethyltransferase T	Amino acid transport and metabolism

lmo1730		10.85	N-Acetyl-D-glucosamine ABC transport system, sugar-binding protein	Carbohydrate transport and metabolism
lmo0879		10.67	Sugar phosphate isomerases/epimerases	Carbohydrate transport and metabolism
lmos72		10.54	No description	
lmo0425		10.48	PRD/PTS system regulatory domain protein	Transcription; Carbohydrate transport and metabolism; Signal transduction mechanism
lmo0678	*fliR*	10.43	Flagellar biosynthesis protein FliR	Cell motility; Intracellular trafficking and secretion
lmo1732		10.39	N-Acetyl-D-glucosamine ABC transport system, permease protein 2	Carbohydrate transport and metabolism
lmo1256		10.19	Hypothetical protein	Replication, recombination and repair; General function prediction only
lmo1046	*moaC*	10.08	Molybdenum cofactor biosynthesis protein MoaC	Coenzyme transport and metabolism
lmo0110		9.96	Esterase/lipase	Lipid transport and metabolism
lmo2799		9.72	PTS system, mannitol-specific IIB component (EC 2.7.1.69) / PTS system, mannitol-specific IIC component	Carbohydrate transport and metabolism
lmo2668		9.65	Predicted galactitol operon regulator (Transcriptional antiterminator), BglG family / PTS system, mannitol/fructose-specific IIA component	Transcription; Carbohydrate transport and metabolism; Signal transduction mechanisms
lmo1998		9.45	Glucosamine—fructose-6-phosphate aminotransferase [isomerizing]	Cell wall/membrane biogenesis
lmo1255		8.69	PTS system, trehalose-specific IIB component / PTS system, trehalose-specific IIC component	Carbohydrate transport and metabolism
lmo2302		8.59	Hypothetical protein	Not in COGs
lmo1060		8.50	Two-component response regulator	Transcription; Signal transduction mechanisms
lmo0691	*cheY*	8.47	Chemotaxis response regulator CheY	Signal transduction mechanisms
lmos39		8.18	No description	
lmo1350		7.98	Glycine dehydrogenase subunit 2	Amino acid transport and metabolism
lmo2665		7.76	PTS system, galactitol-specific IIC component	Carbohydrate transport and metabolism
lmo0765		7.76	Hypothetical protein	Function unknown
lmo2408		7.64	DNA-binding protein	Transcription
lmos81		7.46	No description	
lmo0707	*fliD*	7.45	Flagellar capping protein	Cell motility
lmo2732		7.39	Phosphosugar-binding protein	Transcription
lmo2336	*fruB*	7.38	Fructose-1-phosphate kinase	Carbohydrate transport and metabolism

lmo2160		7.32	Inosose isomerase	Carbohydrate transport and metabolism
lmo2159		7.21	Myo-inositol 2-dehydrogenase	General function prediction only
lmo0769		7.20	Putative alpha-1,6-mannanase	Carbohydrate transport and metabolism
lmo0686	*motB*	7.15	Flagellar motor rotation protein MotB	Cell motility
lmo2685		6.97	PTS system, beta-glucoside-specific IIA component (EC 2.7.1.69); PTS system, cellobiose-specific IIA component	Carbohydrate transport and metabolism
lmo1699		6.95	Methyl-accepting chemotaxis protein	Cell motility; Signal transduction mechanisms
lmo2788	*bvrA*	6.81	Transcription antiterminator	Transcription
lmo0688		6.64	Glycosyl transferase, group 2 family protein	Cell wall/membrane biogenesis; General function prediction only
lmo0866a		6.54	Cold-shock DEAD-box protein A	Replication, recombination and repair; Transcription; Translation
lmo1061		6.54	Hypothetical protein	Signal transduction mechanisms
lmo2443		6.29	Hypothetical protein	Not in COGs
lmo0130		6.27	Hypothetical protein	Nucleotide transport and metabolism
lmo2797		6.23	PTS system, mannitol-specific IIA component	Carbohydrate transport and metabolism; Signal transduction mechanisms
lmo0689		6.11	Chemotaxis protein CheV	Cell motility; Signal transduction mechanisms
lmos05		6.09	No description	
lmo1137		6.05	Hypothetical protein	Function unknown
lmo2180		6.04	Hypothetical protein	Not in COGs
lmo1539		5.99	Glycerol uptake facilitator protein	Carbohydrate transport and metabolism
lmo0049		5.88	Accessory gene regulator protein D, putative	Not in COGs
lmo0692	*cheA*	5.70	Two-component sensor histidine kinase CheA	Cell motility; Signal transduction mechanisms
lmos42		5.63	No description	
lmos33		5.62	No description	
lmo0622		5.56	Hypothetical protein	Not in COGs
lmo2094		5.52	Class II aldolase/adducin domain protein	Carbohydrate transport and metabolism
lmo0474		5.51	Hypothetical protein	Not in COGs
lmo2696		5.44	Phosphoenolpyruvate-dihydroxyacetone phosphotransferase (EC 2.7.1.121), ADP-binding subunit DhaL	Carbohydrate transport and metabolism
lmo2742		5.38	Hypothetical protein	Not in COGs
lmo0677	*fliQ*	5.33	Flagellar biosynthesis protein FliQ	Cell motility; Intracellular trafficking and secretion
Lmo0422	*lstR*	5.30	Lineage-specific thermal regulator protein	Transcription

lmos36		5.24	No description	
lmo0536		5.16	6-phospho-beta-glucosidase	Carbohydrate transport and metabolism
lmo0183		5.13	Alpha-glucosidase	Carbohydrate transport and metabolism
lmo2855	*rnpA*	5.09	Ribonuclease P	Translation
lmo2210		5.09	Hypothetical protein	Not in COGs
lmo0360a		4.95	No description	
lmo2649	*ulaA*	4.83	PTS system ascorbate-specific transporter subunit IIC	Function unknown
lmo2697		4.83	Phosphotransferase mannnose-specific family component IIA	Function unknown
lmo2125		4.77	Maltose/maltodextrin ABC transporter, substrate binding periplasmic protein MalE	Carbohydrate transport and metabolism
lmo1150		4.71	Transcriptional regulator PocR	Transcription; Signal transduction mechanisms
lmo0680	*flhA*	4.70	Flagellar biosynthesis protein FlhA	Cell motility; Intracellular trafficking and secretion
lmo1408		4.67	Transcriptional regulator, PadR family	Transcription
lmo1538	*glpK*	4.65	Glycerol kinase	Energy production and conversion
lmo2831		4.58	Beta-phosphoglucomutase	General function prediction only
lmo2590		4.56	Scaffold protein for [4Fe-4S] cluster assembly ApbC, MRP-like	Cell cycle control, mitosis and meiosis
lmo1665		4.55	Hypothetical protein	Not in COGs
lmo1143		4.42	Propanediol utilization polyhedral body protein PduT	Secondary metabolites biosynthesis, transport and catabolism; Energy production and conversion
lmo2084		4.34	Aminoglycoside phosphotransferase	General function prediction only
lmo1569	*fxsA*	4.32	FxsA	General function prediction only
lmo2857		4.22	Hypothetical protein	Not in COGs
lmo0676	*fliP*	4.20	Flagellar biosynthesis protein FliP	Cell motility; Intracellular trafficking and secretion
lmo2778		4.19	Hypothetical protein	Not in COGs
lmo0306		4.17	Hypothetical protein	Not in COGs
lmo1883		4.16	Chitinase	Carbohydrate transport and metabolism
lmos46		4.02	No description	
lmo1088	*tagB*	3.95	Hypothetical protein	Cell wall/membrane biogenesis
lmo2240		3.91	Hypothetical protein	Defense/virulence mechanisms
lmo2129		3.87	Hypothetical protein	Not in COGs
lmo2187		3.83	Hypothetical protein	Not in COGs

lmo2331		3.82	Hypothetical protein	Not in COGs
lmo2695		3.78	Dihydroxyacetone kinase subunit DhaK	Carbohydrate transport and metabolism
lmo0386		3.76	Hypothetical protein	Amino acid transport and metabolism
lmo2684		3.75	PTS system, cellobiose-specific IIC component	Carbohydrate transport and metabolism
lmo2325a		3.74	No description	
lmo0050		3.63	Histidine kinase of the competence regulon ComD	Signal transduction mechanisms
lmos38		3.63	No description	
lmo2325		3.62	Hypothetical protein	Not in COGs
lmo2709		3.58	Hypothetical protein	Not in COGs
lmo2436		3.50	Hypothetical protein	Transcription
lmo2337		3.50	Transcriptional repressor of the fructose operon, DeoR family	Transcription; Carbohydrate transport and metabolism
lmo0020		3.39	Transcriptional regulator, GntR family	Transcription
lmor01		3.36	No description	
lmo1119		3.35	DNA-methyltransferase	Replication, recombination and repair
lmo0393		3.29	Hypothetical protein	Not in COGs
lmo0436		3.28	Rrf2 family transcriptional regulator, group III	Transcription
lmo0341		3.22	Hypothetical protein	Not in COGs
lmo0471		3.21	Hypothetical protein	Not in COGs
lmo2410		3.21	Hypothetical protein	Not in COGs
lmo1639		3.20	DNA-3-methyladenine glycosylase	Replication, recombination and repair
lmo0212		3.18	Acetyltransferase, GNAT famil	Transcription; General function prediction only
lmo1189		3.16	Transcriptional regulator, AraC family	Transcription
lmos59		3.13	No description	
lmo1188		3.12	Putative major teichoic acid biosynthesis protein C	Not in COGs
lmo2335	*fruA*	3.09	PTS system, fructose-specific IIA component / PTS system, fructose-specific IIB component / PTS system, fructose-specific IIC component	Carbohydrate transport and metabolism; Signal transduction mechanisms
lmo1966		3.07	5-bromo-4-chloroindolyl phosphate hydrolysis protein	General function prediction only
lmo2332	*int*	3.04	Putative integrase	Replication, recombination and repair
lmo0394		3.00	Hypothetical protein	Cell wall/membrane biogenesis
lmo0804		2.99	Hypothetical protein	Not in COGs

lmo0690	*flaA*	2.97	Flagellin	Cell motility
lmos45		2.97	No description	
lmo0391		2.97	Hypothetical protein	Posttranslational modification, protein turnover, chaperones
lmo0496		2.96	Hypothetical protein	Function unknown
lmo2124		2.93	Maltose/maltodextrin ABC transporter, permease protein MalF	Carbohydrate transport and metabolism
lmo0778		2.90	Hypothetical protein	Not in COGs
lmo0475		2.89	Hypothetical protein	Not in COGs
lmo2330		2.89	Protein gp33 [Bacteriophage A118]	Amino acid transport and metabolism
lmo0352		2.89	Transcriptional regulator of rhamnose utilization, DeoR family	Transcription; Carbohydrate transport and metabolism
lmo1173		2.88	Ethanolamine sensory transduction histidine kinase	Signal transduction mechanisms
lmo2369		2.88	General stress protein 13	Translation
lmo0470		2.88	Adenine-specific methyltransferase	Replication, recombination and repair
lmo0983		2.82	Glutathione peroxidase	Posttranslational modification, protein turnover, chaperones
lmo0042		2.81	DedA protein	Function unknown
lmo1097a		2.81	No description	
lmo1097		2.80	Integrase, superantigen-encoding pathogenicity islands SaPI	Replication, recombination and repair
lmo1293	*glpD*	2.77	Aerobic glycerol-3-phosphate dehydrogenase	Energy production and conversion
lmo1867		2.76	Pyruvate phosphate dikinase	Carbohydrate transport and metabolism
lmo0459		2.73	Conserved domain protein	Not in COGs
lmo2707		2.71	Hypothetical protein	Not in COGs
lmo2277		2.68	Hypothetical protein	Nucleotide transport and metabolism
lmo2464		2.65	Hypothetical protein	Transcription
lmo2239		2.64	Membrane protein	Not in COGs
lmo1410		2.64	Hypothetical protein	Not in COGs
lmo1955		2.63	Tyrosine recombinase XerD	Replication, recombination and repair
lmo0388		2.61	Hypothetical protein	Not in COGs
lmo0771		2.61	Hypothetical protein	Not in COGs
lmo2796		2.58	N-acetylmannosamine kinase	Transcription; Carbohydrate transport and metabolism
lmo2275		2.58	Protein gp28	Not in COGs
lmo2273		2.56	Protein gp30	Not in COGs

lmot46		2.56	No description	
lmos57		2.54	No description	
lmo2152		2.54	Thioredoxin, putative	Posttranslational modification, protein turnover, chaperones; Energy production and conversion
lmo0844		2.52	Endoribonuclease L-PSP	Translation
lmo1142		2.51	Hypothetical protein	Energy production and conversion
lmo0671		2.50	Hypothetical protein	Not in COGs
lmo2532	*atpH*	2.49	F0F1 ATP synthase subunit delta	Energy production and conversion
lmo0469		2.49	Hypothetical protein	Not in COGs
lmo0261		2.48	Glycosyl hydrolase, family 1	Carbohydrate transport and metabolism
lmo0816		2.48	Protease synthase and sporulation negative regulatory protein pai 1	Transcription; General function prediction only
lmo0168		2.47	Transition state regulatory protein AbrB	Transcription
lmos75		2.47	No description	
lmo0946		2.47	Hypothetical protein	Not in COGs
lmo1118		2.46	Hypothetical protein	Not in COGs
lmo2218		2.46	Hypothetical protein SAV1840	Not in COGs
lmos43		2.46	No description	
lmo0786		2.45	Acyl-carrier protein phosphodiesterase, putative	Lipid transport and metabolism
lmo0466		2.45	Hypothetical protein	Not in COGs
lmo2245		2.45	Glyoxalase family protein	Amino acid transport and metabolism
lmo2246		2.44	DNA alkylation repair enzyme	Replication, recombination and repair
lmo0101		2.44	Transcriptional regulator, ArsR family	Transcription
lmo1684		2.44	D-3-phosphoglycerate dehydrogenas	Amino acid transport and metabolism; Coenzyme transport and metabolism
lmo0472		2.43	Hypothetical protein	Not in COGs
lmo0606		2.43	Hypothetical protein	Transcription
lmo1900	*panD*	2.43	Aspartate alpha-decarboxylase	Coenzyme transport and metabolism
lmo2349		2.40	L-Cystine ABC transporter, periplasmic cystine-binding protein TcyK	Amino acid transport and metabolism; Signal transduction mechanisms
lmo1340		2.39	Lipoprotein	Not in COGs
lmo0556		2.36	Phosphoglycerate mutase family	Carbohydrate transport and metabolism

lmo1049		2.36	Molybdopterin biosynthesis protein MoeB	Coenzyme transport and metabolism
lmo1802		2.35	Putative DNA-binding protein	Function unknown
lmo2086		2.35	DNA-binding protein	Transcription; General function prediction only
lmo0635		2.35	2-haloalkanoic acid dehalogenase	General function prediction only
lmo0813		2.35	Fructokinase	Transcription; Carbohydrate transport and metabolism
lmo1412		2.34	Modulates DNA topology	Nucleotide transport and metabolism
lmo1913		2.34	Hypothetical protein	Not in COGs
lmo1486		2.33	Lojap protein	Function unknown
lmos13		2.32	No description	
lmo2141		2.30	Acetyltransferase, GNAT family	Transcription; General function prediction only
lmo1893		2.29	Hypothetical protein in cluster with penicillin-binding protein PBP1, Listerial type	Not in COGs
lmo2564		2.27	4-oxalocrotonate tautomerase (EC 5.3.2.-); Xylose transport system permease protein xylH	General function prediction only
lmo2854		2.26	Inner membrane protein translocase component YidC, short form OxaI-like	Intracellular trafficking and secretion
lmo1668		2.26	Hypothetical protein	Not in COGs
lmo1417		2.25	Membrane protein, putative	General function prediction only
lmo2536	*atpI*	2.25	Hypothetical protein	Not in COGs
lmo1994		2.25	Transcriptional regulator, LacI family	Transcription
lmo2743		2.23	Putative translaldolase	Carbohydrate transport and metabolism
lmo2690		2.23	Transcriptional regulator, TetR family	Transcription
lmo1336		2.22	5-formyltetrahydrofolate cyclo-ligase	Coenzyme transport and metabolism
lmo0573		2.22	Xanthine/uracil permease family protein	General function prediction only
lmo1091		2.21	Beta-1,3-glucosyltransferase	Cell wall/membrane biogenesis
lmo2686		2.19	Hypothetical protein	Not in COGs
lmo0278		2.19	Multiple sugar ABC transporter, ATP-binding protein	Carbohydrate transport and metabolism
lmo1116		2.19	Transcriptional regulator, AraC family	Transcription
lmo2334		2.18	DNA-binding protein	Transcription
lmo0478		2.17	Putative secreted protein	Not in COGs
lmo2764		2.17	N-acetylmannosamine kinase	Transcription; Carbohydrate transport and metabolism
lmo1419		2.15	Hypothetical protein	General function prediction only

lmo0030		2.15	Hydrolase, haloacid dehalogenase-like family	General function prediction only
lmo0252		2.15	Transcriptional regulator, putative	Transcription
lmo2197		2.15	Hypothetical protein	Energy production and conversion
lmo2184		2.14	Heme transporter IsdDEF, lipoprotein IsdE	Inorganic ion transport and metabolism
lmo1048		2.14	Molybdenum cofactor biosynthesis protein MoaB	Coenzyme transport and metabolism
lmo1066		2.13	Inositol-1-monophosphatase	Carbohydrate transport and metabolism
lmo2832		2.12	Glycerate kinase	Carbohydrate transport and metabolism
lmo1746		2.12	Hypothetical protein	Defense/virulence mechanisms
lmo2276		2.11	Hypothetical protein	Not in COGs
lmo0588		2.10	Deoxyribodipyrimidine photolyase	Replication, recombination and repair
lmo1878		2.09	Manganese transport transcriptional regulator	Transcription
lmo1954	*drm*	2.09	Phosphopentomutase	Carbohydrate transport and metabolism
lmo2482	*lgt*	2.08	Prolipoprotein diacylglyceryl transferase	Cell wall/membrane biogenesis
lmo2079		2.07	Hypothetical protein	Not in COGs
lmo0281		2.07	Cyclic nucleotide-binding protein	Signal transduction mechanisms
lmo1862		2.07	Lipase/Acylhydrolase with GDSL-like motif	Amino acid transport and metabolism
lmo1953	*pnp*	2.06	Purine nucleoside phosphorylase	Nucleotide transport and metabolism
lmo0637		2.06	2-heptaprenyl-1,4-naphthoquinone methyltransferase	Secondary metabolites biosynthesis, transport and catabolism; General function prediction only
lmo0775		2.06	Hypothetical protein	Not in COGs
lmo0640		2.05	Hypothetical protein	General function prediction only
lmo1707		2.04	Hypothetical protein	Function unknown
lmo0851		2.04	Hypothetical protein	Not in COGs
lmo2165		2.04	Hypothetical protein	Signal transduction mechanisms
lmo2574		2.03	Hypothetical protein	Not in COGs
lmo1333		2.03	Hypothetical protein	General function prediction only
lmo1752		2.03	Hypothetical protein	Not in COGs
lmo2853		2.03	RNA-binding protein Jag	General function prediction only
lmo0407		2.03	Hypothetical protein	Function unknown
lmo1085		2.03	Hypothetical protein	Cell wall/membrane biogenesis
lmo1500		2.02	DedA family protein	Function unknown

lmo1533	*ruvA*	2.02	Holliday junction DNA helicase RuvA	Replication, recombination and repair
lmo2544		2.01	Thymidine kinase	Nucleotide transport and metabolism
lmo1511		2.00	Carboxymethylenebutenolidase-related protein	General function prediction only
lmo1407	*pflC*	2.00	Pyruvate-formate lyase activating enzyme	Posttranslational modification, protein turnover, chaperones
lmo0051		2.00	Response regulator of the competence regulon ComE	Transcription; Signal transduction mechanisms
lmor04		-16.47	No description	
lmo1983	*ilvD*	-15.56	Dihydroxy-acid dehydratase	Amino acid transport and metabolism; Carbohydrate transport and metabolism
lmo1987	*leuA*	-10.13	2-isopropylmalate synthase	Amino acid transport and metabolism
lmor09		-9.14	No description	
lmo2006	*alsS*	-7.85	Acetolactate synthase	Amino acid transport and metabolism; Coenzyme transport and metabolism
lmos95		-7.31	No description	
lmo1989	*leuC*	-7.16	Isopropylmalate isomerase large subunit	Amino acid transport and metabolism
lmo2711		-6.85	Hypothetical protein	Not in COGs
lmor05		-6.52	No description	
lmo1599	*ccpA*	-6.39	Catabolite control protein A	Transcription
lmo1990	*leuD*	-6.23	Isopropylmalate isomerase small subunit	Amino acid transport and metabolism
lmo1991	*ilvA*	-6.19	Threonine dehydratase	Amino acid transport and metabolism
lmo2254		-5.89	Xanthine/uracil/thiamine/ascorbate permease family protein	General function prediction only
lmo2602		-5.11	Hypothetical protein	Function unknown
lmo0097		-5.01	PTS system, mannose-specific IIC component	Carbohydrate transport and metabolism
lmo0098		-4.98	PTS system, mannose-specific IID component	Carbohydrate transport and metabolism
lmo1984	*ilvB*	-4.98	Acetolactate synthase large subunit	Amino acid transport and metabolism; Coenzyme transport and metabolism
lmos74		-4.93	No description	
lmo1986	*ilvC*	-4.77	Ketol-acid reductoisomerase	Amino acid transport and metabolism; Coenzyme transport and metabolism
lmo1985	*ilvH*	-4.64	Acetolactate synthase 3 regulatory subunit	Amino acid transport and metabolism
lmo0263	*inlH*	-4.49	Internalin H	Cell wall/membrane biogenesis
lmo1516		-4.34	Ammonium transporter	Inorganic ion transport and metabolism

lmo0585		-4.23	Putative secreted protein	Not in COGs
lmo0913		-4.23	Succinate-semialdehyde dehydrogenase [NAD] ; Succinate-semialdehyde dehydrogenase [NADP+]	Energy production and conversion
lmos14		-4.00	No description	
lmo0596		-3.79	Hypothetical protein	Function unknown
lmo0134		-3.73	Acetyltransferase, GNAT family	General function prediction only
lmor07		-3.68	No description	
lmot38		-3.65	No description	
lmos24		-3.52	No description	
lmo1799		-3.43	Peptidoglycan binding protein	Not in COGs
lmo2095		-3.35	Tagatose-6-phosphate kinase (EC 2.7.1.144) / 1-phosphofructokinase	Carbohydrate transport and metabolism
lmo2050		-3.34	Excinuclease ABC subunit A paralog of unknown function	Replication, recombination and repair
lmo1015	*gbuB*	-3.27	Glycine betaine ABC transport system, permease protein OpuAB	Amino acid transport and metabolism
lmo0560		-3.24	Glutamate dehydrogenase	Amino acid transport and metabolism
lmo2170		-3.18	Enoyl-[acyl-carrier-protein] reductase [FMN]	General function prediction only
lmo2157	*sepA*	-3.13	Alkyl sulfatase	Secondary metabolites biosynthesis, transport and catabolism
lmo2251		-3.13	Lipid transport and metabolism; Secondary metabolites biosynthesis, transport and catabolism; General function prediction only	Amino acid transport and metabolism
lmo1830		-3.12	Short chain dehydrogenase	Lipid transport and metabolism; Secondary metabolites biosynthesis, transport and catabolism; General function prediction only
lmo2748		-3.12	Inorganic ion transport and metabolism	General function prediction only
lmo0593		-3.02	Formate/nitrite transporter family protein	Inorganic ion transport and metabolism
lmo0994		-3.01	Hypothetical protein	Not in COGs
lmo1992		-2.96	Alpha-acetolactate decarboxylase	Secondary metabolites biosynthesis, transport and catabolism
lmo0055	*purA*	-2.94	Adenylosuccinate synthetase	Nucleotide transport and metabolism
lmo2829		-2.92	Nitroreductase family protein	General function prediction only
lmo0283		-2.90	Methionine ABC transporter permease protein	Inorganic ion transport and metabolism
lmo0782		-2.85	PTS system, mannose-specific IIC component / PTS system, fructose-specific IIC component	Carbohydrate transport and metabolism

lmo0554		-2.85	NADH-dependent butanol dehydrogenase A	Energy production and conversion
lmo1364	*cspL*	-2.83	Cold-shock protein	Transcription
lmo1767	*purM*	-2.80	Phosphoribosylaminoimidazole synthetase	Nucleotide transport and metabolism
lmo2230		-2.80	Arsenate reductase	Signal transduction mechanisms
lmo0538		-2.80	Catalyzes the cleavage of p-aminobenzoyl-glutamate to p-aminobenzoate and glutamate, subunit A	General function prediction only
lmo1787	*rplS*	-2.79	50S ribosomal protein L19	Translation
lmo2340		-2.78	Pseudouridine 5'-phosphate glycosidase	Secondary metabolites biosynthesis, transport and catabolism
lmo2827		-2.78	Hypothetical protein	Transcription
lmo0722		-2.77	Pyruvate oxidase	Amino acid transport and metabolism; Coenzyme transport and metabolism
lmo2626	*rpsC*	-2.77	30S ribosomal protein S3	Translation
lmos93		-2.76	No description	
lmo2249		-2.74	Probable low-affinity inorganic phosphate transporter	Inorganic ion transport and metabolism
lmo0757		-2.69	ABC transporter, permease protein	General function prediction only
lmo0781		-2.67	PTS system mannose-specific transporter subunit IID	Carbohydrate transport and metabolism
lmos53		-2.67	No description	
lmo1330	*rpsO*	-2.64	30S ribosomal protein S15	Translation
lmo1425	*opuCD*	-2.63	Osmotically activated L-carnitine/choline ABC transporter, permease protein OpuCD	Amino acid transport and metabolism
lmo1428	*opuCA*	-2.61	Osmotically activated L-carnitine/choline ABC transporter, ATP-binding protein OpuCA	Amino acid transport and metabolism
lmo2653	*tuf*	-2.60	Elongation factor Tu	Translation
lmo2729		-2.60	Transcriptional regulator	Function unknown
lmor13		-2.58	No description	
lmo1011		-2.56	2,3,4,5-tetrahydropyridine-2,6-dicarboxylate N-acetyltransferase	Amino acid transport and metabolism
lmo0458		-2.52	N-methylhydantoinase (ATP-hydrolyzing	Amino acid transport and metabolism; Secondary metabolites biosynthesis, transport and catabolism
lmo2689a		-2.51	No description	
lmo0794		-2.49	Rrf2-linked NADH-flavin reductase	General function prediction only
lmo0210	*Ldh*	-2.49	L-lactate dehydrogenase	Energy production and conversion
lmo2657		-2.48	Deoxyguanosinetriphosphate triphosphohydrolase-like protein	Nucleotide transport and metabolism

lmo1769	*purQ*	-2.47	Phosphoribosylformylglycinamidine synthase II	Nucleotide transport and metabolism
lmo1765	*purH*	-2.46	Bifunctional phosphoribosylaminoimidazolecarboxamide formyltransferase/IMP cyclohydrolase	Nucleotide transport and metabolism
lmo2463		-2.45	Hypothetical protein	General function prediction only
lmo2398	*ltrC*	-2.44	Low temperature requirement C protein	Lipid transport and metabolism
lmo1775	*purE*	-2.43	Phosphoribosylaminoimidazole carboxylase catalytic subunit	Nucleotide transport and metabolism
lmo1596	*rpsD*	-2.42	30S ribosomal protein S4	Translation
lmo1571	*pfkA*	-2.41	6-phosphofructokinase	Carbohydrate transport and metabolism
lmo0537		-2.41	Allantoate amidohydrolase	Amino acid transport and metabolism
lmo2605	*rplQ*	-2.38	50S ribosomal protein L17	Translation
lmo0293		-2.38	rRNA large subunit methyltransferase	Function unknown
lmo2455	*eno*	-2.37	Phosphopyruvate hydratase	Carbohydrate transport and metabolism
lmo2158		-2.34	Hypothetical protein	Function unknown
lmo0783		-2.33	PTS system, mannose-specific IIB component	Carbohydrate transport and metabolism
lmo1570	*pykA*	-2.32	Pyruvate kinase	Carbohydrate transport and metabolism; Signal transduction mechanisms
lmo0648		-2.32	Magnesium and cobalt transport protein CorA	Inorganic ion transport and metabolism
lmo2459	*gap*	-2.31	Glyceraldehyde-3-phosphate dehydrogenase	Carbohydrate transport and metabolism
lmo0539		-2.31	Tagatose 1,6-diphosphate aldolase	Carbohydrate transport and metabolism
lmo0534		-2.31	Hypothetical protein	Function unknown
lmo0907		-2.31	Phosphoglycerate mutase family, Lmo0907 homolog	Carbohydrate transport and metabolism
lmo1622		-2.30	YjeF protein, C-terminal domain	Carbohydrate transport and metabolism
lmo1016	*gbuC*	-2.29	Glycine betaine ABC transport system, glycine betaine-binding protein OpuAC	Amino acid transport and metabolism
lmo0841		-2.29	Cation-transporting ATPase	Inorganic ion transport and metabolism
lmo2749		-2.28	Para-aminobenzoate synthase, amidotransferase component	Amino acid transport and metabolism; Coenzyme transport and metabolism
lmo2654	*fus*	-2.27	Elongation factor G	Translation
lmo2596	*rpsI*	-2.27	30S ribosomal protein S9	Translation
lmo1540	*rpmA*	-2.22	50S ribosomal protein L27	Translation
lmo0532		-2.22	Hypothetical protein	Secondary metabolites biosynthesis, transport and catabolism; General function prediction only

lmo1993	*pdp*	-2.21	Pyrimidine-nucleoside phosphorylase	Nucleotide transport and metabolism
lmo1764	*purD*	-2.20	Phosphoribosylamine--glycine ligase	Nucleotide transport and metabolism
glnA	*glnA*	-2.20	Glutamine synthetase type I	Amino acid transport and metabolism
lmo0099		-2.19	Putative regulator of the mannose operon, ManO	Function unknown
lmo0137		-2.19	Hypothetical protein	Amino acid transport and metabolism; Inorganic ion transport and metabolism
lmo0831		-2.18	Hypothetical protein	General function prediction only
lmo0551		-2.18	Extracellular protein	Not in COGs
lmo2250	*arpJ*	-2.18	Amino acid ABC transporter, amino acid-binding/permease protein	Amino acid transport and metabolism; Signal transduction mechanisms
lmot30		-2.18	No description	
lmo2687		-2.17	Cell division protein FtsW	Cell cycle control, mitosis and meiosis
lmo1839	*pyrP*	-2.17	Uracil permease	Nucleotide transport and metabolism
lmo0814		-2.16	Enoyl-[acyl-carrier-protein] reductase [FMN]	General function prediction only
lmo0512		-2.15	Hypothetical protein	Not in COGs
lmo2692		-2.13	Protein from nitrogen regulatory protein P-II (GLNB) family, ortholog YAAQ B. subtilis	Function unknown
lmo0990		-2.12	Multi antimicrobial extrusion protein (Na(+)/drug antiporter), MATE family of MDR efflux pumps	Defense/virulence mechanisms
lmo2101		-2.12	Pyridoxal biosynthesis lyase PdxS	Coenzyme transport and metabolism
lmo1936	*gpsA*	-2.12	NAD(P)H-dependent glycerol-3-phosphate dehydrogenase	Energy production and conversion
lmo2366		-2.11	Transcriptional regulator of rhamnose utilization, DeoR family	Transcription; Carbohydrate transport and metabolism
lmo2389		-2.10	NADH dehydrogenase	Energy production and conversion
lmo2556	*fbaA*	-2.09	Fructose-bisphosphate aldolase class II	Carbohydrate transport and metabolism
lmo0796		-2.09	Hypothetical protein	Function unknown
lmo0208		-2.09	Virulence cluster protein B VclB	Function unknown
lmo0724		-2.08	Hypothetical protein	Function unknown
lmo2615	*rpsE*	-2.07	30S ribosomal protein S5	Translation
lmot29		-2.07	No description	
lmo1664	*metK*	-2.07	S-adenosylmethionine synthetase	Coenzyme transport and metabolism
lmo1796		-2.07	KH domain RNA binding protein YlqC	General function prediction only

lmo2205		-2.07	Phosphoglycerate mutase	Carbohydrate transport and metabolism
lmo0269		-2.07	Oligopeptide transport system permease protein OppC	Amino acid transport and metabolism; Inorganic ion transport and metabolism
lmo0845		-2.06	Methionine synthase II (cobalamin-independent)	Amino acid transport and metabolism
lmo2405		-2.05	Hypothetical protein	Function unknown
lmo1978		-2.04	Glucose-6-phosphate 1-dehydrogenase	Carbohydrate transport and metabolism
lmo0197		-2.04	Regulatory protein SpoVG	Cell wall/membrane biogenesis
lmo0866		-2.04	Cold-shock DEAD-box protein A	Replication, recombination and repair; Transcription; Translation;
lmo0793		-2.03	Putative transport protein	General function prediction only
lmo2049		-2.03	Hypothetical protein	General function prediction only
lmo0314		-2.03	Cellobiose phosphotransferase system YdjC-like protein	Function unknown
lmo0201	*plcA*	-2.03	Phosphatidylinositol-specific phospholipase c	Defense/virulence mechanisms
lmo2622	*rplN*	-2.02	50S ribosomal protein L14	Translation
lmo0161		-2.02	STAS domain protein	Signal transduction mechanisms
lmo1846		-2.01	Multidrug efflux protein	Defense/virulence mechanisms
lmo1483	*comEB*	-2.01	Hypothetical protein	Nucleotide transport and metabolism

[1] *lmo* denotes a protein coding gene; *lmor* a gene encoding a non-coding ribosomal RNA; *lmos* a gene encoding a non-coding RNA product that cannot be defined by other RNA keys (also known as miscRNA), and *lmot* a non-coding transfer RNA.

[2] Information from Listeriomics website (https://listeriomics.pasteur.fr/Listeriomics/#bacnet.Listeria). If only Rapid Annotations using Subsystems Technology (RAST)-products of the gene was found, RAST-products are listed as Description.

Supplementary Table S5: Genes ≥ 2.0-fold up-regulated and ≤ -2.0-fold down-regulated ($p \leq 0.05$) in *Listeria monocytogenes prfA**-Δ*ccpA* compared to *prfA** upon exposure to 10µg/mL LA for 1hour.

Gene symbol[1]	Gene name	Fold change	Description[2]	Clusters of Orthologous Genes (COGs)[2]
lmo2772		397.52	PTS system, beta-glucoside-specific IIB component / PTS system, beta-glucoside-specific IIC component / PTS system, beta-glucoside-specific IIA component	Carbohydrate transport and metabolism
lmo2771		274.01	6-phospho-beta-glucosidase	Carbohydrate transport and metabolism
lmo0517		169.11	Hypothetical, related to broad specificity phosphatases COG0406	Carbohydrate transport and metabolism
lmo2851		160.93	Transcriptional regulator of rhamnose utilization, AraC family	Transcription
lmo1879	*cspD*	97.16	Cold shock protein CspB	Transcription
lmo2797		89.69	PTS system, mannitol-specific IIA component	Carbohydrate transport and metabolism; Signal transduction mechanisms
lmo1730		74.06	N-Acetyl-D-glucosamine ABC transport system, sugar-binding protein	Carbohydrate transport and metabolism
lmo2585		72.96	Hypothetical protein YrhD	Function unknown
lmo0025		62.67	Hypothetical protein	General function prediction only
lmo2586		52.30	Formate dehydrogenase related protein	General function prediction only
lmo1731		51.50	Hypothetical protein YrhD	Function unknown
lmo0027		51.34	PTS system, beta-glucoside-specific IIB component / PTS system, beta-glucoside-specific IIC component / PTS system, beta-glucoside-specific IIA component	Carbohydrate transport and metabolism
lmo0682	*flgG*	49.34	Flagellar basal body rod protein FlgG	Cell motility
lmo2584		48.97	Formate dehydrogenase accessory protein	Energy production and conversion
lmo0024		46.70	Hypothetical protein	Carbohydrate transport and metabolism
lmo2708		44.97	PTS system, cellobiose-specific IIC component	Carbohydrate transport and metabolism
lmo0678	*fliR*	43.65	Flagellar biosynthesis protein FliR	Cell motility; Intracellular trafficking and secretion
lmo0023		40.51	PTS system, mannose-specific IIC component	Carbohydrate transport and metabolism
lmo1045		37.87	Molybdenum cofactor biosynthesis protein MoaD	Coenzyme transport and metabolism
lmo2163		36.79	Myo-inositol 2-dehydrogenase 1	General function prediction only
lmo2668		34.21	Predicted galactitol operon regulator (Transcriptional antiterminator), BglG family / PTS system, mannitol/fructose-specific IIA component	Transcription; Carbohydrate transport and metabolism; Signal transduction mechanisms
lmo1043		33.83	Molybdopterin-guanine dinucleotide biosynthesis protein MobB	Coenzyme transport and metabolism
lmo2667		32.79	PTS system, galactitol-specific IIA component	Carbohydrate transport and metabolism; Signal transduction mechanisms
lmo0859		31.80	Multiple sugar ABC transporter, substrate-binding protein	Carbohydrate transport and metabolism

lmo0508		31.07	PTS system, galactitol-specific IIC component	Carbohydrate transport and metabolism
lmo2773		30.62	Beta-glucoside bgl operon antiterminator, BglG family	Transcription
lmo2409		30.45	Hypothetical protein	Not in COGs
lmo2649	*ulaA*	30.01	PTS system ascorbate-specific transporter subunit IIC	Function unknown
lmo0357		29.99	PTS system, fructose-specific IIA component	Carbohydrate transport and metabolism; Signal transduction mechanisms
lmo2527		28.60	Hypothetical protein	Function unknown
lmo2002		26.65	PTS system, mannose-specific IIB component	Carbohydrate transport and metabolism
lmo0701		26.53	Hypothetical protein	Not in COGs
lmo2800		26.18	Oxidoreductase, Gfo/Idh/MocA family	General function prediction only
lmo0699	*fliM*	24.49	Flagellar motor switch protein FliM	Cell motility
lmo1042		24.19	Molybdopterin biosynthesis protein MoeA	Coenzyme transport and metabolism
lmo2175	*fabG*	23.47	3-ketoacyl-(acyl-carrier-protein) reductase	Lipid transport and metabolism; Secondary metabolites biosynthesis, transport and catabolism; General function prediction only
lmo0109		22.82	Transcriptional regulator, AraC family	Transcription
lmo0685		22.49	Flagellar motor protein MotA	Cell motility
lmo0862		21.26	Trehalose-6-phosphate hydrolase	Carbohydrate transport and metabolism
lmo1254		21.21	Trehalose-6-phosphate hydrolase	Carbohydrate transport and metabolism
lmo0643		21.07	Hypothetical protein	Carbohydrate transport and metabolism
lmo0686	*motB*	21.00	Flagellar motor rotation protein MotB	Cell motility
lmo0718		21.00	Hypothetical protein	Not in COGs
lmo0543		20.80	PTS system, glucitol/sorbitol-specific IIB component and second of two IIC components	Carbohydrate transport and metabolism
lmo0372		20.70	Beta-glucosidase	Carbohydrate transport and metabolism
lmo0323		19.71	Endonuclease/exonuclease/phosphatase family protein	General function prediction only
lmo1700		18.84	Hypothetical protein	Not in COGs
lmo2662		18.73	Ribose 5-phosphate isomerase B	Carbohydrate transport and metabolism
lmo0105		18.48	Hypothetical protein	Carbohydrate transport and metabolism; General function prediction only
lmo2099		18.17	Predicted galactitol operon regulator (Transcriptional antiterminator), BglG family / PTS system, mannitol/fructose-specific IIA component	Transcription; Carbohydrate transport and metabolism; Signal transduction mechanisms
lmo0032		17.63	Sugar kinase and transcription regulator	Transcription; Carbohydrate transport and metabolism
lmo1346	*comGB*	17.47	Late competence protein ComGB, access of DNA to ComEA	Cell motility; Intracellular trafficking and secretion
lmo2007		17.41	Multiple sugar ABC transporter, substrate-binding protein	Carbohydrate transport and metabolism
lmo0876		17.09	Hypothetical protein	Carbohydrate transport and metabolism

lmo0022		16.83	PTS system, mannose-specific IIB component / PTS system, mannose-specific IIA component	Carbohydrate transport and metabolism
lmo2798		16.55	Hydrolase, haloacid dehalogenase-like family	General function prediction only
lmo2003		16.50	Transcriptional regulator, GntR family	Transcription
lmo2651		16.47	PTS system, IIA component	Carbohydrate transport and metabolism; Signal transduction mechanisms
lmo0110		16.31	Esterase/lipase	Lipid transport and metabolism
lmo0425		16.28	PRD/PTS system regulatory domain protein	Transcription; Carbohydrate transport and metabolism; Signal transduction mechanism
lmo0713	*fliF*	16.09	Flagellar MS-ring protein	Cell motility; Intracellular trafficking and secretion
lmo0707	*fliD*	15.73	Flagellar capping protein	Cell motility
lmos25		15.33	No description	
lmo0879		15.03	Sugar phosphate isomerases/epimerases	Carbohydrate transport and metabolism
lmo2850		14.96	Predicted L-rhamnose permease RhaY	Carbohydrate transport and metabolism; Amino acid transport and metabolism; Inorganic ion transport and metabolism; General function prediction only
lmo1348	*gcvT*	14.92	Glycine cleavage system aminomethyltransferase T	Amino acid transport and metabolism
lmo0915		14.88	PTS system, cellobiose-specific IIC component	Carbohydrate transport and metabolism
lmo0681		14.66	Flagellar biosynthesis regulator FlhF	Cell motility
lmo1191	*cbiA*	14.58	Cobyrinic acid a,c-diamide synthase	Coenzyme transport and metabolism
lmo0917		14.44	6-phospho-beta-glucosidase	Carbohydrate transport and metabolism
lmo2665		14.44	PTS system, galactitol-specific IIC component	Carbohydrate transport and metabolism
lmo1047	*moaA*	14.22	Molybdenum cofactor biosynthesis protein A	Coenzyme transport and metabolism
lmo0692	*cheA*	13.32	Two-component sensor histidine kinase CheA	Cell motility; Signal transduction mechanisms
lmo0021		13.11	PTS system, IIA component	Carbohydrate transport and metabolism
lmo0776		12.83	Fructokinase	Transcription; Carbohydrate transport and metabolism
lmo1255		12.73	PTS system, trehalose-specific IIB component / PTS system, trehalose-specific IIC component	Carbohydrate transport and metabolism
lmos31		12.65	No description	
lmos81		12.35	No description	
lmo2161		11.87	Hypothetical protein	Carbohydrate transport and metabolism
lmo2799		11.69	PTS system, mannitol-specific IIB component (EC 2.7.1.69) / PTS system, mannitol-specific IIC component	Carbohydrate transport and metabolism
lmo1349		11.66	Glycine dehydrogenase subunit 1	Amino acid transport and metabolism
lmo2787	*bvrB*	11.53	Beta-glucoside-specific phosphotransferase enzyme II ABC component	Carbohydrate transport and metabolism
lmo0697	*flgE*	11.42	Flagellar hook protein FlgE	Cell motility

lmo0680	*flhA*	10.94	Flagellar biosynthesis protein FlhA	Cell motility; Intracellular trafficking and secretion
lmo2683		10.89	PTS system, cellobiose-specific IIB component	Carbohydrate transport and metabolism
lmo0675		10.87	Flagellar motor switch protein FliN	Not in COGs
lmo1999		10.23	Glucosamine--fructose-6-phosphate aminotransferase [isomerizing]	Cell wall/membrane biogenesis
lmo0679	*flhB*	9.71	Flagellar biosynthesis protein FlhB	Cell motility; Intracellular trafficking and secretion
lmo1350		9.65	Glycine dehydrogenase subunit 2	Amino acid transport and metabolism
lmos34		9.40	No description	
lmos42		9.40	No description	
lmo1997		9.25	PTS system, mannose-specific IIA component	Carbohydrate transport and metabolism
lmo0688		8.80	Glycosyl transferase, group 2 family protein	Cell wall/membrane biogenesis; General function prediction only
lmo2685		8.79	PTS system, beta-glucoside-specific IIA component; PTS system, cellobiose-specific IIA component	Carbohydrate transport and metabolism
lmo1044		8.73	Molybdenum cofactor biosynthesis protein MoaE	Coenzyme transport and metabolism
lmo2336	*fruB*	8.57	Fructose-1-phosphate kinase	Carbohydrate transport and metabolism
lmo1998		8.34	Glucosamine--fructose-6-phosphate aminotransferase [isomerizing]	Cell wall/membrane biogenesis
lmo1699		8.32	Methyl-accepting chemotaxis protein	Cell motility; Signal transduction mechanisms
lmo2664		8.24	Galactitol-1-phosphate 5-dehydrogenase	Amino acid transport and metabolism; General function prediction only
lmot41		8.16	No description	
lmo0878		8.06	Oxidoreductase, aldo/keto reductase family	Energy production and conversion
lmo1256		7.70	Hypothetical protein	Replication, recombination and repair; General function prediction only
lmo2786	*bvrC*	7.58	ADP-ribosylglycohydrolase YegU	Posttranslational modification, protein turnover, chaperones
lmo1539		7.52	Glycerol uptake facilitator protein	Carbohydrate transport and metabolism
lmo2443		7.40	Hypothetical protein	Not in COGs
lmo2159		7.35	Myo-inositol 2-dehydrogenase	General function prediction only
lmo0130		7.31	Hypothetical protein	Nucleotide transport and metabolism
lmo2788	*bvrA*	7.29	Transcription antiterminator	Transcription
lmo2125		7.23	Maltose/maltodextrin ABC transporter, substrate binding periplasmic protein MalE	Carbohydrate transport and metabolism
lmo2408		7.21	DNA-binding protein	Transcription
lmo0639		7.16	Transcriptional regulator	Not in COGs
lmo1150		6.97	Transcriptional regulator PocR	Transcription; Signal transduction mechanisms
lmos48		6.52	No description	

lmo1338		6.50	Hypothetical protein	Function unknown
lmo0705	*flgK*	6.48	Flagellar hook-associated protein FlgK	Cell motility
lmo0689		6.27	Chemotaxis protein CheV	Cell motility; Signal transduction mechanisms
lmo2684		6.25	PTS system, cellobiose-specific IIC component	Carbohydrate transport and metabolism
lmo2742		6.22	Hypothetical protein	Not in COGs
lmo1046	*moaC*	5.96	Molybdenum cofactor biosynthesis protein MoaC	Coenzyme transport and metabolism
lmo0536		5.77	6-phospho-beta-glucosidase	Carbohydrate transport and metabolism
lmo1883		5.46	Chitinase	Carbohydrate transport and metabolism
lmo0474		5.43	Hypothetical protein	Not in COGs
lmo2697		5.35	Phosphotransferase mannnose-specific family component IIA	Function unknown
lmo2696		4.94	Phosphoenolpyruvate-dihydroxyacetone phosphotransferase (EC 2.7.1.121), ADP-binding subunit DhaL	Carbohydrate transport and metabolism
lmo0278		4.80	Multiple sugar ABC transporter, ATP-binding protein	Carbohydrate transport and metabolism
lmo0676	*fliP*	4.78	Flagellar biosynthesis protein FliP	Cell motility; Intracellular trafficking and secretion
lmo1538	*glpK*	4.63	Glycerol kinase	Energy production and conversion
lmo2590		4.61	Scaffold protein for [4Fe-4S] cluster assembly ApbC, MRP-like	Cell cycle control, mitosis and meiosis
lmo2187		4.49	Hypothetical protein	Not in COGs
lmo2796		4.43	N-acetylmannosamine kinase	Transcription; Carbohydrate transport and metabolism
lmo0049		4.39	Accessory gene regulator protein D, putative	Not in COGs
lmo2831		4.39	Beta-phosphoglucomutase	General function prediction only
lmo2695		4.30	Dihydroxyacetone kinase subunit DhaK	Carbohydrate transport and metabolism
lmo0358		4.26	PTS system, fructose-specific IIBC component (EIIBC-Fru) (Fructose- permease IIBC component) (Phosphotransferase enzyme II, BC component)	Carbohydrate transport and metabolism
lmo2160		4.23	Inosose isomerase	Carbohydrate transport and metabolism
lmo1293	*glpD*	4.21	Aerobic glycerol-3-phosphate dehydrogenase	Energy production and conversion
lmo2337		4.19	Transcriptional repressor of the fructose operon, DeoR family	Transcription; Carbohydrate transport and metabolism
lmo0815		3.88	Transcriptional regulator, MarR family	Transcription
lmo2335	*fruA*	3.81	PTS system, fructose-specific IIA component / PTS system, fructose-specific IIB component / PTS system, fructose-specific IIC component	Carbohydrate transport and metabolism; Signal transduction mechanisms
lmo1097a		3.67	No description	
lmo0677	*fliQ*	3.62	Flagellar biosynthesis protein FliQ	Cell motility; Intracellular trafficking and secretion
lmo1049		3.62	Molybdopterin biosynthesis protein MoeB	Coenzyme transport and metabolism
lmo2436		3.51	Hypothetical protein	Transcription
lmos45		3.51	Hypothetical protein	Not in COGs
lmo2709		3.48	Hypothetical protein	Not in COGs

lmos33		3.48	No description	
lmo2123		3.44	Maltose/maltodextrin ABC transporter, permease protein MalG	Carbohydrate transport and metabolism
lmo2743		3.36	Putative translaldolase	Carbohydrate transport and metabolism
lmo2129		3.28	Hypothetical protein	Not in COGs
lmo2857		3.28	Hypothetical protein	Not in COGs
lmo2855	*rnpA*	3.25	Ribonuclease P	Translation
lmo0690	*flaA*	3.21	Flagellin	Cell motility
lmo1867		3.12	Pyruvate phosphate dikinase	Carbohydrate transport and metabolism
lmos46		3.05	No description	
lmo1732		3.05	N-Acetyl-D-glucosamine ABC transport system, permease protein 2	Carbohydrate transport and metabolism
lmo1188		3.04	Putative major teichoic acid biosynthesis protein C	Not in COGs
lmo2180		3.01	Hypothetical protein	Not in COGs
lmo0816		3.00	Protease synthase and sporulation negative regulatory protein pai 1	Transcription; General function prediction only
lmos59		2.93	No description	
lmo0261		2.87	Glycosyl hydrolase, family 1	Carbohydrate transport and metabolism
lmo1569	*fxsA*	2.85	FxsA	General function prediction only
lmo0471		2.80	Hypothetical protein	Not in COGs
lmo1061		2.79	Hypothetical protein	Signal transduction mechanisms
lmo0386		2.77	Hypothetical protein	Amino acid transport and metabolism
lmos38		2.77	No description	
lmo0983		2.75	Glutathione peroxidase	Posttranslational modification, protein turnover, chaperones
lmo2659		2.74	Ribulose-phosphate 3-epimerase	Carbohydrate transport and metabolism
lmo0478		2.73	Putative secreted protein	Not in COGs
lmo1097		2.73	Integrase, superantigen-encoding pathogenicity islands SaPI	Replication, recombination and repair
lmo2369		2.72	General stress protein 13	Translation
lmo1955		2.72	Tyrosine recombinase XerD	Replication, recombination and repair
lmo0260		2.71	Hypothetical protein	General function prediction only
lmo2210		2.69	Hypothetical protein	Not in COGs
lmo0394		2.62	Hypothetical protein	Cell wall/membrane biogenesis
lmo0020		2.60	Transcriptional regulator, GntR family	Transcription
lmo0393		2.59	Hypothetical protein	Not in COGs
lmo0813		2.57	Fructokinase	Transcription; Carbohydrate transport and metabolism
lmo1410		2.56	Hypothetical protein	Not in COGs
lmo2832		2.50	Glycerate kinase	Carbohydrate transport and metabolism

lmo0178		2.50	Putative ROK-family transcriptional regulator	Transcription; Carbohydrate transport and metabolism
lmo1119		2.48	DNA-methyltransferase	Replication, recombination and repair
lmo0606		2.48	Hypothetical protein	Transcription
lmo2532	*atpH*	2.46	F0F1 ATP synthase subunit delta	Energy production and conversion
lmo0252		2.46	Transcriptional regulator, putative	Transcription
lmo0050		2.39	Histidine kinase of the competence regulon ComD	Signal transduction mechanisms
lmo0429		2.36	Alpha-mannosidase	Carbohydrate transport and metabolism
lmo0212		2.35	Acetyltransferase, GNAT famil	Transcription; General function prediction only
lmo0640		2.34	Hypothetical protein	General function prediction only
lmo1954	*drm*	2.33	Phosphopentomutase	Carbohydrate transport and metabolism
lmo2277		2.33	Hypothetical protein	Nucleotide transport and metabolism
lmo1048		2.32	Molybdenum cofactor biosynthesis protein MoaB	Coenzyme transport and metabolism
lmo1917	*pflA*	2.30	Pyruvate formate-lyase	Energy production and conversion
lmo1028		2.29	Protein of unknown function	Function unknown
lmo2464		2.25	Hypothetical protein	Transcription
lmo1408		2.25	Transcriptional regulator, PadR family	Transcription
lmo1802		2.24	Putative DNA-binding protein	Function unknown
lmo0771		2.21	Hypothetical protein	Not in COGs
lmo1665		2.18	Hypothetical protein	Not in COGs
lmo2331		2.18	Hypothetical protein	Not in COGs
lmo2349		2.17	L-Cystine ABC transporter, periplasmic cystine-binding protein TcyK	Amino acid transport and metabolism; Signal transduction mechanisms
lmo2424		2.16	Hypothetical protein	Posttranslational modification, protein turnover, chaperones; Energy production and conversion
lmo0557		2.15	Phosphoglycerate mutase family 1	Carbohydrate transport and metabolism
lmo0469		2.14	Hypothetical protein	Not in COGs
lmo0775		2.14	Hypothetical protein	Not in COGs
lmo0786		2.13	Acyl-carrier protein phosphodiesterase, putative	Lipid transport and metabolism
lmo2334		2.12	DNA-binding protein	Transcription
lmo0042		2.12	DedA protein	Function unknown
lmo1717		2.12	Hypothetical protein	Function unknown
lmos43		2.11	No description	
lmo0388		2.10	Hypothetical protein	Not in COGs
lmo1953	*pnp*	2.09	Purine nucleoside phosphorylase	Nucleotide transport and metabolism
lmo2246		2.08	DNA alkylation repair enzyme	Replication, recombination and repair
lmo2239		2.05	Membrane protein	Not in COGs

lmo2582		2.02	Sensor histidine kinase colocalized with HrtAB transporter	Signal transduction mechanisms
lmo2853		2.01	RNA-binding protein Jag	General function prediction only
lmo2707		2.01	Hypothetical protein	Not in COGs
lmo0472		2.01	Hypothetical protein	Not in COGs
lmo1419		2.00	Hypothetical protein	General function prediction only
lmo1988	*leuB*	-10.23	3-isopropylmalate dehydrogenase	Energy production and conversion; Amino acid transport and metabolism
lmor04		-8.81	No description	
lmo1599	*ccpA*	-8.38	Catabolite control protein A	Transcription
lmo2006	*alsS*	-7.68	Acetolactate synthase	Amino acid transport and metabolism; Coenzyme transport and metabolism
lmo1983	*ilvD*	-7.44	Dihydroxy-acid dehydratase	Amino acid transport and metabolism; Carbohydrate transport and metabolism
lmo1987	*leuA*	-7.01	2-isopropylmalate synthase	Amino acid transport and metabolism
lmo1991	*ilvA*	-5.90	Threonine dehydratase	Amino acid transport and metabolism
lmo1989	*leuC*	-5.74	Isopropylmalate isomerase large subunit	Amino acid transport and metabolism
lmor05		-5.35	No description	
lmor09		-5.25	No description	
lmo1984	*ilvB*	-5.14	Acetolactate synthase large subunit	Amino acid transport and metabolism; Coenzyme transport and metabolism
lmo1986	*ilvC*	-5.01	Ketol-acid reductoisomerase	Amino acid transport and metabolism; Coenzyme transport and metabolism
lmo2254		-4.91	Xanthine/uracil/thiamine/ascorbate permease family protein	General function prediction only
lmos95		-4.50	No description	
lmo2711		-4.28	Hypothetical protein	Not in COGs
lmos74		-4.02	No description	
lmo1992		-3.54	Alpha-acetolactate decarboxylase	Secondary metabolites biosynthesis, transport and catabolism
lmo1768	*purF*	-3.49	Amidophosphoribosyltransferase	Nucleotide transport and metabolism
lmo1799		-3.05	Peptidoglycan binding protein	Not in COGs
lmo0593		-3.03	Formate/nitrite transporter family protein	Inorganic ion transport and metabolism
lmo1775	*purE*	-3.02	Phosphoribosylaminoimidazole carboxylase catalytic subunit	Nucleotide transport and metabolism
lmo0098		-2.90	PTS system, mannose-specific IID component	Carbohydrate transport and metabolism
lmo0439		-2.86	Siderophore/Surfactin synthetase related protein	General function prediction only
lmo2829		-2.83	Nitroreductase family protein	General function prediction only
lmo1015	*gbuB*	-2.81	Glycine betaine ABC transport system, permease protein OpuAB	Amino acid transport and metabolism
lmo0055	*purA*	-2.80	Adenylosuccinate synthetase	Nucleotide transport and metabolism
lmo2050		-2.75	Excinuclease ABC subunit A paralog of unknown function	Replication, recombination and repair

lmot38		-2.74	No description	
lmo2095		-2.71	Tagatose-6-phosphate kinase / 1-phosphofructokinase	Carbohydrate transport and metabolism
lmo0263	*inlH*	-2.70	Internalin H	Cell wall/membrane biogenesis
lmo0560		-2.66	Glutamate dehydrogenase	Amino acid transport and metabolism
lmor07		-2.66	No description	
lmo0210	*ldh*	-2.57	L-lactate dehydrogenase	Energy production and conversion
lmo1769	*purQ*	-2.57	Phosphoribosylformylglycinamidine synthase II	Nucleotide transport and metabolism
lmo2249		-2.56	Probable low-affinity inorganic phosphate transporter	Inorganic ion transport and metabolism
lmos53		-2.49	No description	
lmo0596		-2.46	Hypothetical protein	Function unknown
lmo0097		-2.45	PTS system, mannose-specific IIC component	Carbohydrate transport and metabolism
lmo2251		-2.44	Amino acid ABC transporter, ATP-binding protein	Amino acid transport and metabolism
lmo1974		-2.38	Transcriptional regulator, GntR family, putative	Transcription
lmo2157	*sepA*	-2.37	Alkyl sulfatase	Secondary metabolites biosynthesis, transport and catabolism
lmo2821		-2.37	Internalin-like protein (LPXTG motif)	Cell wall/membrane biogenesis
lmo0538		-2.30	Catalyzes the cleavage of p-aminobenzoyl-glutamate to p-aminobenzoate and glutamate, subunit A	General function prediction only
lmo0794		-2.29	Rrf2-linked NADH-flavin reductase	General function prediction only
lmo2748		-2.25	General stress protein 26	General function prediction only
lmo1764	*purD*	-2.24	Phosphoribosylamine--glycine ligase	Nucleotide transport and metabolism
lmo2340		-2.24	Pseudouridine 5'-phosphate glycosidase	Secondary metabolites biosynthesis, transport and catabolism
lmo1839	*pyrP*	-2.23	Uracil permease	Nucleotide transport and metabolism
lmo1570	*pykA*	-2.19	pyruvate kinase	Carbohydrate transport and metabolism; Signal transduction mechanisms
lmor13		-2.18	No description	
lmo2749		-2.17	Para-aminobenzoate synthase, amidotransferase component	Amino acid transport and metabolism; Coenzyme transport and metabolism
lmo2588		-2.16	Lincomycin resistance protein LmrB	Carbohydrate transport and metabolism; Amino acid transport and metabolism; Inorganic ion transport and metabolism; General function prediction only
lmo0539		-2.10	Tagatose 1,6-diphosphate aldolase	Carbohydrate transport and metabolism
lmo1765	*purH*	-2.08	Bifunctional phosphoribosylaminoimidazolecarboxamide formyltransferase/IMP cyclohydrolase	Nucleotide transport and metabolism
lmo1016	*gbuC*	-2.07	Glycine betaine ABC transport system, glycine betaine-binding protein OpuAC	Amino acid transport and metabolism
lmos71		-2.05	No description	
lmo1622		-2.05	YjeF protein, C-terminal domain	Carbohydrate transport and metabolism

lmo2049		-2.04	hypothetical protein	Not in COGs
lmo1596	*rpsD*	-2.04	30S ribosomal protein S4	Translation
lmo1787	*rplS*	-2.03	50S ribosomal protein L19	Translation
lmo1299	*glnA*	-2.03	Glutamine synthetase type I	Amino acid transport and metabolism
lmo2653	*tuf*	-2.03	Elongation factor Tu	Translation
lmo2248		-2.02	Phosphate transport regulator (distant homolog of PhoU)	Inorganic ion transport and metabolism
lmo2114		-2.02	Bacitracin export ATP-binding protein BceA	Defense/virulence mechanisms
lmo2115		-2.02	Bacitracin export permease protein BceB	Defense/virulence mechanisms
lmo0973	*dltB*	-2.00	DltB protein for D-alanine esterification of lipoteichoic acid and wall teichoic acid	Cell wall/membrane biogenesis

[1] *lmo* denotes a protein coding gene; *lmor* a gene encoding a non-coding ribosomal RNA; *lmos* a gene encoding a non-coding RNA product that cannot be defined by other RNA keys (also known as miscRNA), and *lmot* a non-coding transfer RNA.

[2] Information from Listeriomics website (https://listeriomics.pasteur.fr/Listeriomics/#bacnet.Listeria). If only Rapid Annotations using Subsystems Technology (RAST)-products of the gene was found, RAST-products are listed as Description.

Supplementary Table S6: Genes ≥ 4.0-fold up-regulated and ≤ -4.0-fold down-regulated ($p \leq 0.05$) in *Listeria monocytogenes prfA**-Δ*ccpA* compared to *prfA** both during growth in BHI ("Control fold change") and upon exposure to 10µg/mL LA for 1hour ("LA fold change").

Gene symbol[1]	Gene name	Description[2]	Clusters of Orthologous Genes (COGs)[2]	Control fold change	LA fold change
lmo2772		PTS system, beta-glucoside-specific IIB component / PTS system, beta-glucoside-specific IIC component / PTS system, beta-glucoside-specific IIA component	Carbohydrate transport and metabolism	269.40	397.52
lmo2771		6-phospho-beta-glucosidase	Carbohydrate transport and metabolism	279.49	274.01
lmo0517		Hypothetical, related to broad specificity phosphatases	Carbohydrate transport and metabolism	186.85	169.11
lmo2851		Transcriptional regulator of rhamnose utilization, AraC family	Transcription	17.59	160.93
lmo1879	*cspD*	Cold shock protein CspB	Transcription	93.80	97.16
lmo2797		PTS system, mannitol-specific IIA component	Carbohydrate transport and metabolism; Signal transduction mechanisms	6.23	89.69
lmo1730		N-Acetyl-D-glucosamine ABC transport system, sugar-binding protein	Carbohydrate transport and metabolism	10.85	74.06
lmo2585		Hypothetical protein YrhD	Function unknown	59.15	72.96
lmo0025		Hypothetical protein	General function prediction only	37.08	62.67
lmo2586		Formate dehydrogenase related protein	General function prediction only	45.58	52.30
lmo1731		Hypothetical protein YrhD	Function unknown	49.10	51.50
lmo0027		PTS system, beta-glucoside-specific IIB component / PTS system, beta-glucoside-specific IIC component / PTS system, beta-glucoside-specific IIA component	Carbohydrate transport and metabolism	62.60	51.34
lmo0682	*flgG*	Flagellar basal body rod protein FlgG	Cell motility	12.64	49.34
lmo2584		Formate dehydrogenase accessory protein	Energy production and conversion	32.58	48.97
lmo0024		Hypothetical protein	Carbohydrate transport and metabolism	41.67	46.70
lmo2708		PTS system, cellobiose-specific IIC component	Carbohydrate transport and metabolism	17.14	44.97
lmo0678	*fliR*	Flagellar biosynthesis protein FliR	Cell motility; Intracellular trafficking and secretion	10.43	43.65
lmo0023		PTS system, mannose-specific IIC component	Carbohydrate transport and metabolism	28.73	40.51
lmo1045		Molybdenum cofactor biosynthesis protein MoaD	Coenzyme transport and metabolism	20.24	37.87
lmo2163		Myo-inositol 2-dehydrogenase 1	General function prediction only	24.28	36.79
lmo2668		Predicted galactitol operon regulator (Transcriptional antiterminator), BglG family / PTS system, mannitol/fructose-specific IIA component	Transcription; Carbohydrate transport and metabolism; Signal transduction mechanisms	9.65	34.21

lmo1043		Molybdopterin-guanine dinucleotide biosynthesis protein MobB	Coenzyme transport and metabolism	22.92	33.83
lmo0859		Multiple sugar ABC transporter, substrate-binding protein	Carbohydrate transport and metabolism	12.65	31.80
lmo0508		PTS system, galactitol-specific IIC component	Carbohydrate transport and metabolism	12.80	31.07
lmo2773		Beta-glucoside bgl operon antiterminator, BglG family	Transcription	26.19	30.62
lmo2409		Hypothetical protein	Not in COGs	29.38	30.45
lmo2649	*ulaA*	PTS system ascorbate-specific transporter subunit IIC	Function unknown	4.83	30.01
lmo0357		PTS system, fructose-specific IIA component	Carbohydrate transport and metabolism; Signal transduction mechanisms	30.17	30.00
lmo1042		Molybdopterin biosynthesis protein MoeA	Coenzyme transport and metabolism	25.49	24.19
lmo2175	*fabG*	3-ketoacyl-(acyl-carrier-protein) reductase	Lipid transport and metabolism; Secondary metabolites biosynthesis, transport and catabolism; General function prediction only	10.98	23.47
lmo0109		Transcriptional regulator, AraC family	Transcription	31.67	22.82
lmo0685		Flagellar motor protein MotA	Cell motility	20.20	22.48
lmo0862		Trehalose-6-phosphate hydrolase	Carbohydrate transport and metabolism	13.24	21.26
lmo1254		Trehalose-6-phosphate hydrolase	Carbohydrate transport and metabolism	18.66	21.21
lmo0643		Hypothetical protein	Carbohydrate transport and metabolism	40.45	21.07
lmo0686	*motB*	Flagellar motor rotation protein MotB	Cell motility	7.15	21.00
lmo0718		Hypothetical protein	Not in COGs	16.42	20.99
lmo0543		PTS system, glucitol/sorbitol-specific IIB component and second of two IIC components	Carbohydrate transport and metabolism	12.72	20.80
lmo0323		Endonuclease/exonuclease/phosphatase family protein	General function prediction only	15.15	19.71
lmo0105		Hypothetical protein	Carbohydrate transport and metabolism; General function prediction only	35.31	18.48
lmo2099		Predicted galactitol operon regulator (Transcriptional antiterminator), BglG family / PTS system, mannitol/fructose-specific IIA component	Transcription; Carbohydrate transport and metabolism; Signal transduction mechanisms	12.41	18.17
lmo0022		PTS system, mannose-specific IIB component / PTS system, mannose-specific IIA component	Carbohydrate transport and metabolism	15.86	16.83
lmo2798		Hydrolase, haloacid dehalogenase-like family	General function prediction only	67.76	16.55
lmo2651		PTS system, IIA component	Carbohydrate transport and metabolism; Signal transduction mechanisms	13.27	16.47
lmo0110		Esterase/lipase	Lipid transport and metabolism	9.96	16.31

lmo0425		PRD/PTS system regulatory domain protein	Transcription; Carbohydrate transport and metabolism; Signal transduction mechanism	10.48	16.28
lmo0713	*fliF*	Flagellar MS-ring protein	Cell motility; Intracellular trafficking and secretion	11.51	16.09
lmo0707	*fliD*	Flagellar capping protein	Cell motility	7.45	15.73
lmos25		No description		19.77	15.33
lmo0879		Sugar phosphate isomerases/epimerases	Carbohydrate transport and metabolism	10.67	15.03
lmo1348	*gcvT*	Glycine cleavage system aminomethyltransferase T	Amino acid transport and metabolism	10.88	14.92
lmo0681		Flagellar biosynthesis regulator FlhF	Cell motility	79.03	14.66
lmo2665		PTS system, galactitol-specific IIC component	Carbohydrate transport and metabolism	7.76	14.44
lmo1047	*moaA*	Molybdenum cofactor biosynthesis protein A	Coenzyme transport and metabolism	12.16	14.22
lmo0692	*cheA*	Two-component sensor histidine kinase CheA	Cell motility; Signal transduction mechanisms	5.70	13.32
lmo0021		PTS system, IIA component	Carbohydrate transport and metabolism	19.04	13.11
lmo0776		Fructokinase	Transcription; Carbohydrate transport and metabolism	13.22	12.83
lmo1255		PTS system, trehalose-specific IIB component / PTS system, trehalose-specific IIC component	Carbohydrate transport and metabolism	8.69	12.73
lmos31		No description		19.48	12.65
lmos81		No description		7.46	12.35
lmo2799		PTS system, mannitol-specific IIB component / PTS system, mannitol-specific IIC component	Carbohydrate transport and metabolism	9.72	11.69
lmo1349		Glycine dehydrogenase subunit 1	Amino acid transport and metabolism	10.90	11.66
lmo2787	*bvrB*	Beta-glucoside-specific phosphotransferase enzyme II ABC component	Carbohydrate transport and metabolism	69.07	11.53
lmo0697	*flgE*	Flagellar hook protein FlgE	Cell motility	34.04	11.42
lmo0680	*flhA*	Flagellar biosynthesis protein FlhA	Cell motility; Intracellular trafficking and secretion	4.70	10.94
lmo2683		PTS system, cellobiose-specific IIB component	Carbohydrate transport and metabolism	28.40	10.89
lmo0675		Flagellar motor switch protein FliN	Not in COGs	20.51	10.87
lmo1999		Glucosamine--fructose-6-phosphate aminotransferase [isomerizing]	Cell wall/membrane biogenesis	26.22	10.23
lmo0679	*flhB*	Flagellar biosynthesis protein FlhB	Cell motility; Intracellular trafficking and secretion	13.65	9.71
lmo1350		Glycine dehydrogenase subunit 2	Amino acid transport and metabolism	7.98	9.65
lmos34		No description		14.40	9.40

lmos42		No description		5.63	9.40
lmo1997		PTS system, mannose-specific IIA component	Carbohydrate transport and metabolism	34.66	9.25
lmo0688		Glycosyl transferase, group 2 family protein	Cell wall/membrane biogenesis; General function prediction only	6.64	8.80
lmo2685		PTS system, beta-glucoside-specific IIA component; PTS system, cellobiose-specific IIA component	Carbohydrate transport and metabolism	6.97	8.79
lmo1044		Molybdenum cofactor biosynthesis protein MoaE	Coenzyme transport and metabolism	13.53	8.73
lmo2336	*fruB*	Fructose-1-phosphate kinase	Carbohydrate transport and metabolism	7.38	8.57
lmo1998		Glucosamine--fructose-6-phosphate aminotransferase [isomerizing]	Cell wall/membrane biogenesis	9.45	8.34
lmo1699		Methyl-accepting chemotaxis protein	Cell motility; Signal transduction mechanisms	6.95	8.32
lmot41		No description		12.62	8.16
lmo1256		Hypothetical protein	Replication, recombination and repair; General function prediction only	10.19	7.70
lmo2786	*bvrC*	ADP-ribosylglycohydrolase YegU	Posttranslational modification, protein turnover, chaperones	16.04	7.58
lmo1539		Glycerol uptake facilitator protein	Carbohydrate transport and metabolism	5.99	7.52
lmo2443		Hypothetical protein	Not in COGs	6.29	7.40
lmo2159		Myo-inositol 2-dehydrogenase	General function prediction only	7.21	7.35
lmo0130		Hypothetical protein	Nucleotide transport and metabolism	6.27	7.31
lmo2788	*bvrA*	Transcription antiterminator	Transcription	6.81	7.29
lmo2125		Maltose/maltodextrin ABC transporter, substrate binding periplasmic protein MalE	Carbohydrate transport and metabolism	4.77	7.23
lmo2408		DNA-binding protein	Transcription	7.64	7.21
lmo1150		Transcriptional regulator PocR	Transcription; Signal transduction mechanisms	4.71	6.97
lmos48		No description		15.72	6.52
lmo1338		Hypothetical protein	Function unknown	19.58	6.50
lmo0689		Chemotaxis protein CheV	Cell motility; Signal transduction mechanisms	6.11	6.27
lmo2742		Hypothetical protein	Not in COGs	5.38	6.22
lmo1046	*moaC*	Molybdenum cofactor biosynthesis protein MoaC	Coenzyme transport and metabolism	10.08	5.96
lmo0536		6-phospho-beta-glucosidase	Carbohydrate transport and metabolism	5.16	5.77
lmo1883		Chitinase	Carbohydrate transport and metabolism	4.16	5.46
lmo0474		Hypothetical protein	Not in COGs	5.51	5.43

lmo2697		Phosphotransferase mannnose-specific family component IIA	Function unknown	4.83	5.35
lmo2696		Phosphoenolpyruvate-dihydroxyacetone phosphotransferase, ADP-binding subunit DhaL	Carbohydrate transport and metabolism	5.44	4.94
lmo0676	*fliP*	Flagellar biosynthesis protein FliP	Cell motility; Intracellular trafficking and secretion	4.20	4.78
lmo1538	*glpK*	Glycerol kinase	Energy production and conversion	4.65	4.63
lmo2590		Scaffold protein for [4Fe-4S] cluster assembly ApbC, MRP-like	Cell cycle control, mitosis and meiosis	4.56	4.61
lmo0049		Accessory gene regulator protein D, putative	Not in COGs	5.88	4.39
lmo2831		Beta-phosphoglucomutase	General function prediction only	4.58	4.39
lmo2160		Inosose isomerase	Carbohydrate transport and metabolism	7.32	4.23
lmor04		No description		-16.47	-8.81
lmo1599	*ccpA*	Catabolite control protein A	Transcription	-6.39	-8.38
lmo2006	*alsS*	Acetolactate synthase	Amino acid transport and metabolism; Coenzyme transport and metabolism	-7.85	-7.68
lmo1983	*ilvD*	Dihydroxy-acid dehydratase	Amino acid transport and metabolism; Carbohydrate transport and metabolism	-15.56	-7.44
lmo1987	*leuA*	2-isopropylmalate synthase	Amino acid transport and metabolism	-10.13	-7.01
lmo1991	*ilvA*	Threonine dehydratase	Amino acid transport and metabolism	-6.19	-5.90
lmo1989	*leuC*	Isopropylmalate isomerase large subunit	Amino acid transport and metabolism	-7.16	-5.74
lmor05		No description		-6.52	-5.35
lmor09		No description		-9.14	-5.25
lmo1984	*ilvB*	Acetolactate synthase large subunit	Amino acid transport and metabolism; Coenzyme transport and metabolism	-4.98	-5.14
lmo1986	*ilvC*	Ketol-acid reductoisomerase	Amino acid transport and metabolism; Coenzyme transport and metabolism	-4.77	-5.01
lmo2254		Xanthine/uracil/thiamine/ascorbate permease family protein	General function prediction only	-5.89	-4.91
lmos95		No description		-7.31	-4.50
lmo2711		Hypothetical protein	Not in COGs	-6.85	-4.28
lmos74		No description		-4.93	-4.02

[1] *lmo* denotes a protein coding gene; *lmor* a gene encoding a non-coding ribosomal RNA; *lmos* a gene encoding a non-coding RNA product that cannot be defined by other RNA keys (also known as miscRNA), and *lmot* a non-coding transfer RNA.

[2] Information from Listeriomics website (https://listeriomics.pasteur.fr/Listeriomics/#bacnet.Listeria). If only Rapid Annotations using Subsystems Technology (RAST)-products of the gene was found, RAST-products are listed as Description.

Supplementary Table S7: Primers used in this study.

Name	Sequence (5'→3')	Further information
**Cloning**		
P1 *ccpA*-mut1	GGGGGAGCTCGGTTACAAAGAAGCATTAGAAGAA	Forward primer for the upstream region of the *ccpA*-mut1 mutation. SacI restriction enzyme site is underlined.
P2 *ccpA*-mut1	AAAATGCTGTCAACTTA**TTTTTTT**CTTTAGTCGTACC	Reverse primer for the upstream region of the *ccpA*-mut1 mutation. $A_8 \rightarrow A_7$ *ccpA*-mut1 mutation is presented in bold.
P3 *ccpA*-mut1	GGTACGACTAAAG**AAAAAAA**TAAGTTGACAGCATTTT	Forward primer for the downstream region of the *ccpA*-mut1. $A_8 \rightarrow A_7$ *ccpA*-mut1 mutation is presented in bold.
P4 *ccpA*-mut1	CCCCTCTAGACCGATATGCATCGAGTCTCC	Reverse primer for the downstream region of the *ccpA*-mut1. XbaI restriction enzyme site is underlined.
P5 *ccpA*-mut1	GCAGTGAAACGTTTTGTAGATAATGGT	Forward primer for validation of chromosomal *ccpA*-mut1 mutation.
P6 *ccpA*-mut1	CCAATAGTTCCTGTCGCTCC	Reverse primer for validation of chromosomal *ccpA*-mut1 mutation.
P1 Δ*ccpA*	GGGGGAGCTCGCAGACGGTGTAATGGCTG	Forward primer for the upstream flanking region of *ccpA*. SacI restriction enzyme site is underlined.
P2 Δ*ccpA*	TACATTCATCTCTATTCACTCTCC	Reverse primer for the upstream flanking region of *ccpA*.

P3 Δ*ccpA*	**GGAGAGTGAATAGAGATGAATGTA**ACTAAAGAAAAAAAATAAGTTGACAGC	Forward primer for the downstream flanking region of *ccpA*. Anneals with P2 Δ*ccpA* presented in bold.
P4 Δ*ccpA*	CCCCTCTAGACTGTTTTTCTTCTACCCATTTGCG	Forward primer for the downstream flanking region of *ccpA*. XbaI restriction enzyme site is underlined.
P5 Δ*ccpA*	GGTTGACGTGACGCATTC	Forward primer for validation of chromosomal deletion of *ccpA*.
P1 Δ*lmo0109-0110*	GGGGGAGCTCCAAGTCTTCCCGAATTCGGAAC	Forward primer for the upstream flanking region of the *lmo0109-lmo0110* operon. SacI restriction enzyme site is underlined.
P2 Δ*lmo0109-0110*	CGTATTTATTTTCAGCATCACTTC	Reverse primer for the upstream flanking region of the *lmo0109-lmo0110* operon.
P3 Δ*lmo0109-0110*	**GAAGTGATGCTGAAAATAAATACG**GAAATATTTTAAAGAATAATTTGCAGGC	Forward primer for the downstream flanking region of the *lmo0109-lmo0110* operon. Anneals with P2 Δ*lmo0109-lmo0110* presented in bold.
P4 Δ*lmo0109-0110*	CCCCGGATCCCTCGCTTTTTAATTTGTCTAGCC	Forward primer for the downstream flanking region of the *lmo0109-lmo0110* operon. BamHI restriction enzyme site is underlined.
P5 Δ*lmo0109-0110*	GCGCCATAATATCCCCAACG	Forward primer for validation of chromosomal deletion of the *lmo0109-lmo0110* operon.
P6 Δ*lmo0109-0110*	CGTTTATGACCGGGTACATCG	Reverse primer for validation of chromosomal deletion of the *lmo0109-lmo0110* operon.
P1 Δ*lmo0517*	GGGGGAGCTCGGTTTGCTGAAACTGTGCAG	Forward primer for the upstream flanking region of *lmo0517*. SacI restriction enzyme site is underlined.

P2 Δ*lmo0517*	ATTTTTCTTCATCTTGTTCTCC	Reverse primer for the upstream flanking region of *lmo0517*.
P3 Δ*lmo0517*	**GGAGAACAAGATGAAGAAAAAT**GGTTCTAAATAAATAACCAGACC	Forward primer for the downstream flanking region of *lmo0517*. Anneals with P2 Δ*lmo0517* presented in bold.
P4 Δ*lmo0517*	CCCCGGATCCCGTATACGATTTTACAGCTTC	Forward primer for the downstream flanking region of *lmo0517*. BamHI restriction enzyme site is underlined.
P5 Δ*lmo0517*	GACGCTATTTGCGAATCAAGGG	Forward primer for validation of chromosomal deletion of *lmo0517*.
P6 Δ*lmo0517*	CAACAGCCGCCATAATAGCTG	Reverse primer for validation of chromosomal deletion of *lmo0517.*
P1 Δ*lmo2772*	GGGGGAGCTCGCGGAAGTAGATAGTGATATG	Forward primer for the upstream flanking region of *lmo2772*. SacI restriction enzyme site is underlined.
P2 Δ*lmo2772*	GTAGTCCATGCTAATCCTCCTA	Reverse primer for the upstream flanking region of *lmo2772*.
P3 Δ*lmo2772*	**TAGGAGGATTAGCATGGACTAC**GAGCAAAATGAGTAATACGAAG	Forward primer for the downstream flanking region of *lmo2772*. Anneals with P2 Δ*lmo2772* presented in bold.
P4 Δ*lmo2772*	CCCCGGATCCGTTCACCTCATTTGTTAATGC	Forward primer for the downstream flanking region of *lmo2772*. BamHI restriction enzyme site is underlined.
P5 Δ*lmo2772*	CAGGAAGAATATGCAGTGGCC	Forward primer for validation of chromosomal deletion of *lmo2772*.
P6 Δ*lmo2772*	CCTCCGCATGACGAATCCC	Reverse primer for validation of chromosomal deletion of *lmo2772.*
P1 Δ*lmo2175*	GGGGGAATTCGGCTTGATGATGATACACATGA	Forward primer for the upstream flanking region of *lmo2175*. EcoRI restriction enzyme site is underlined.
P2 Δ*lmo2175*	CCCCATTGTTATTTCCTCCTTC	Reverse primer for the upstream flanking region of *lmo2175*.

P3 Δ*lmo2175*	**GAAGGAGGAAATAACAATGGGG**ACAGCTTACTAATAAAAATTTCGTCC	Forward primer for the downstream flanking region of *lmo2175*. Anneals with P2 Δ*lmo2175* presented in bold.
P4 Δ*lmo2175*	CCCCGGATCCGCCCACCATAAAAGCCTACC	Forward primer for the downstream flanking region of *lmo2175*. BamHI restriction enzyme site is underlined.
P5 Δ*lmo2175*	CACAAACCTGATTTTGAAAACTGG	Forward primer for validation of chromosomal deletion of *lmo2175*.
P6 Δ*lmo2175*	CAACAGCCGCCATAATAGCTG	Reverse primer for validation of chromosomal deletion of *lmo2175*.
pAUL-1	ATGATTACCGCCCAAGCTTG	Forward primer for validation of plasmid construct
pAUL-2	CAGGACGTTGTAAAACGACG	Reverse primer for validation of plasmid construct
**NB probes**		
lmo2772	CCGTAGTCTGGTAATGCAGTGATAAACTTC	Single stranded probe for *lmo2772* mRNA
lmo0110	CTGTTTAAAAATAACTTCGGTTCCTTCTTCTTGTTC	Single stranded probe for *lmo0110* mRNA
lmo0517	ACCGCAACCTGCAATTAATAGTACTGCGCA	Single stranded probe for *lmo0517* mRNA
lmo2175	CCATTCCAGATGCCGCACCAGTTAC	Single stranded probe for *lmo2175* mRNA
16S	GGCCATTACCCTACCAACTAGCTAATGCAC	Single stranded probe for *16S* mRNA
**Bacterial identification**		

hly forward	GAAGCAAAGGATGCATCTGC	Forward primer for identification of *hly*
hly reverse	CCATCTTTGTAACCTTTTCTTGG	Reverse primer for identification of *hly*
